# Reflections on the Human Genome Diversity Project: a conversation with Marcus W. Feldman, Henry T. Greely, and Mary-Claire King

**DOI:** 10.1093/genetics/iyaf273

**Published:** 2026-02-12

**Authors:** Sohini Ramachandran, Noah A Rosenberg

**Affiliations:** Department of Ecology, Evolution and Organismal Biology, Brown University, Providence, RI 02912, United States; Data Science Institute, Brown University, Providence, RI 02912, United States; Department of Biology, Stanford University, Stanford, CA 94305, United States

**Keywords:** human genetic variation, Human Genome Diversity Project, Human Genome Project, population genetics

## Abstract

The Human Genome Diversity Project (HGDP) began in 1991 as an initiative to study genetic variation from human populations worldwide. In 2002, the HGDP reported the HGDP-CEPH Human Genome Diversity Cell Line Panel, a global panel of 1064 cell lines that is maintained at the Centre d’Etude du Polymorphisme Humain (CEPH) and that has served as a major resource fundamental to the last 25 years of progress in human population genetics. HGDP-CEPH data have been central to research on topics such as human genetic diversity, human population structure, human migrations, the development of population-genetic statistics and software, and the potential value of inclusion of diverse sets of human populations in biomedical research. In this article, two researchers who participated in early analyses of genotypes from the HGDP-CEPH panel in the early 2000s speak with three researchers who played key roles in developing the Human Genome Diversity Project from its origin in 1991. The conversation reflects on the successes and challenges of the effort to launch the HGDP and on its scientific contributions.

## Introduction

Anyone seeking to understand the last 25 years of progress in human evolutionary genetics will quickly encounter findings obtained with the HGDP-CEPH Human Genome Diversity Cell Line Panel (see Appendix). Since 2002, this resource has been indispensable in studies of human genetic diversity, human evolutionary history, population structure, and the geographic distribution of genetic variation. It has been the basis for assessments of global patterns in numerous genes and in genetic marker sets of numerous types. It regularly supplies the data for developing and testing population-genetic statistics and software. Its data have helped motivate the importance of diverse worldwide populations in human biomedical genetics. It has inspired many new forms of visualization of the complexity of patterns of genetic variation. Its samples underlie various subsequent panels and data sets, and they have even been referenced in a Nobel Prize.

When the first genetic data emerged from the HGDP-CEPH panel beginning in 2002, we were among the junior scientists tasked with performing the early data analyses. One of us (N.A.R.) was a recent PhD graduate of Marc Feldman's laboratory with a research focus on statistical analysis of population-genetic data; the other (S.R.) was a Feldman Lab undergraduate, soon to be continuing in the lab as a PhD student.

As we understood during our training in the Feldman lab, the idea of an organized project for global sampling and analysis of human genome diversity had been proposed in 1991 by a team headed by Marc's close colleague, distinguished geneticist L. Luca Cavalli-Sforza, shortly after the official launch of the Human Genome Project in 1990. Although it was broadly supported by human population geneticists and by some anthropologists, the effort that came to be known as the Human Genome Diversity Project (HGDP) had attracted criticism, notably from activists, indigenous groups, bioethicists, and other anthropologists, and it never secured significant government funding. Nevertheless, dozens of researchers used funds from their own labs to create a unique collective resource, contributing to a lymphoblastoid cell line repository that would be made available for human population genetics research and data generation as genomic technologies advanced in the coming decades. The Centre d’Étude du Polymorphisme Humain (CEPH) provided crucial support for the project, agreeing to host the 1064 cell lines and to distribute DNA samples from the cells. Later, the resource became known as the “HGDP-CEPH Human Genome Diversity Cell Line Panel.”

During our scientific training and our early years as independent researchers, we experienced the early heyday of research activity based on the HGDP-CEPH Human Genome Diversity Cell Line Panel, beginning in 2002. At the time that it was introduced, the HGDP-CEPH panel was a rare human population-genetic resource that included many global regions, with multiple populations within regions, and it quickly became a touchstone to address questions of human evolutionary genetics, population-genetic theory, and statistical methods in population genetics more generally (summarized in the Appendix).

Having been too early in our careers to have followed the beginning of the HGDP in real time, we had limited knowledge of the deliberations and challenges of its early years, when the project was proposed and then scaled back from the original vision. We had, however, heard hints that the early events of the project had more dimensions than typical brief recountings of the project would suggest. Curious about the events that led to the development of an important resource for the field (and for our own research programs), we approached three leaders of the HGDP—Marc Feldman, Hank Greely, and Mary-Claire King—about the possibility of an oral history interview. In preparation for the interview, we examined the voluminous documents generated by the early years of the HGDP: among them, meeting reports and other writings from investigators working on the project, contemporaneous news articles, advocacy documents against the project, and scholarship in bioethics and science studies covering the project and the issues it raised. For synthetic accounts written from a variety of perspectives, see the news series by Salopek (1997a,b) that received a Pulitzer Prize for explanatory journalism, a bioethics discussion paper by Resnik (1999) and its 20 responses in *Politics in the Life Sciences*, a book-length treatment in the social studies of science by Reardon (2005) as well as an earlier article by Reardon (2001), a project-adjacent ethnography of human genetic diversity science by M’Charek (2005), perspectives from within the project by Greely (2001) and Cavalli-Sforza (2005), and the Cavalli-Sforza biography by Stone and Lurquin (2005); many additional documents are referenced from the endnotes in our Supplementary Material.

In the development of the HGDP, Marc, Hank, Mary-Claire, and the rest of the HGDP team were early in facing questions that we can now see as intrinsic to the field of human population genetics to this day. What does it mean to try to sample the world's human genetic diversity? What are the proper roles of individuals and populations in the design and analysis of human population-genetic studies? What are appropriate ways for researchers to interact with study subjects beyond formal regulatory requirements? What special considerations are important in genetic research involving indigenous populations? What is the role of human population genetics in contributing to biomedical advances? What types of responses from scientists and scientific institutions are appropriate when they are challenged in ways that do not follow the norms of scientific debate? These questions still resonate; as Hank said to us in relation to such topics, “no good problems ever get solved.” Marc, Hank, and Mary-Claire have long been renowned in the human population genetics field for their humanistic perspective and attention to ethical conduct of research; their efforts, and the successes, challenges, and approaches in the HGDP history more broadly, can serve as a case example through which today's human population geneticists can engage with difficult questions that they are likely to encounter. For the historical record of the HGDP, it is important to hear key project researchers reflect on it in their own words.

Last July, we sat down with Marc, Hank, and Mary-Claire on the Stanford campus to consider the long span of the HGDP, from the original ambitious effort beginning in 1991 to the report of the cell line panel in 2002 to the applications of the panel in subsequent decades and the lessons of the efforts to the launch the project—their thoughts about its early years, its contribution to science, and how they see the project now.

Our wide-ranging conversation traversed many of the central questions that the HGDP faced. The conversation also linked early events of the HGDP to the 1990s era in which they took place—for example, the science funding environment during the Clinton administration, and the elevated attention given at the time to the patenting of biological materials. The rapid rise in electronic communication was a backdrop to the period, facilitating the HGDP's international collaboration via email with coordinator Jean Doble at Stanford; however, it was also portentously noted in one news article (Taubes 1995) critical of HGDP's persistent antagonist, the activist group RAFI (Rural Advancement Foundation International), that the emerging internet could be used not only for rapid communication among scientists, but also for rapid dissemination of misinformation about their activities.

The early history of the HGDP has often been viewed through the lens of the interactions between the scientific world and indigenous populations and activists, and a reader examining the early HGDP documents in 2025 may well be taken aback by the statements that set the criticism of the project in motion, namely the project's references to vanishing populations and disappearing languages. In our conversation, Marc, Hank, and Mary-Claire argued that the HGDP—together with the criticisms it received—ultimately served to advance values that the project had originally sought to promote, such as inclusion of diverse populations, including indigenous populations, and respect for those populations, in human genetic studies.

A modern reader will also notice in early HGDP documents scientific statements that foreshadowed much of the agenda of subsequent research in human population genetics. For us, therefore, a poignant and previously underappreciated thread in the conversation was a sense in which Marc, Hank, and Mary-Claire viewed the fate of HGDP not solely in terms of interactions between scientists and indigenous populations, but also in terms of its unsuccessful effort to gain funding support from the National Center for Human Genome Research (NCHGR), later to become the National Human Genome Research Institute (NHGRI), which was running the Human Genome Project (HGP). The HGDP thus also serves as a case study for issues of the dynamics of the funding of science, the interaction between major projects developed by self-organized groups of scientists and those organized centrally by funders, and the relationship of basic research—in this case on human evolutionary history—to science that possesses an explicitly biomedical motivation.

One of the main events in the story of the federally funded HGP was the intense rivalry it experienced with Craig Venter and Celera Genomics when Venter duplicated the genome sequencing project with a parallel effort. However, when NHGRI, rather than supporting the genetic variation project that was already being developed by many of the leading population geneticists, designed its own separate major project on genetic variation, eventually to become the International Haplotype Map Project, it acted toward the HGDP in the same manner that had caused NHGRI such consternation when Craig Venter and Celera sought to supersede the HGP. Notably, although the HGDP was widely known at the time, the first call from NHGRI for a major study of human DNA variation did not acknowledge it (Collins et al. 1997).

Unlike Venter's genome project, HGDP was entirely noncommercial, and when NIH funds did not materialize, the scaled-down version of HGDP in the form of the HGDP-CEPH collaboration proceeded as a self-organized community project with contributions from many investigators, in contrast to funder-driven initiatives such as the HapMap project, 1000 Genomes Project, and Human Pangenome Project. The HGDP follows a pattern seen in studies of business innovation, in which the first effort to propose an idea can face the first-mover difficulties of uncovering the challenges inherent in the enterprise—but then serves to facilitate subsequent efforts (e.g. Lieberman and Montgomery 1988). The conversation revealed to us the sense of importance Marc, Hank, and Mary-Claire felt for the pioneering role of the project, their pride in the results that were achieved and the values that were advanced, and their admiration and respect for the colleagues who participated in the project, especially Luca.

An edited version of the conversation follows. A timeline of selected major events in the early history of the HGDP appears in Table 1. Other events mentioned in the conversation appear in Table 2.

**Table 1.** Selected events in the history of the Human Genome Diversity Project.

Date	Event
1991	News story by Roberts (1991) describes the upcoming article of Cavalli-Sforza et al. (1991) (1991 June 21)
1991	Letter to the editor by Weiss (1991) in response to Roberts (1991) expresses interest by an NSF program officer in the ideas proposed by Cavalli-Sforza et al. (1991) (1991 September 27)
1991	Publication by Cavalli-Sforza, Wilson, Cantor, Cook-Deegan, and King (Cavalli-Sforza et al. 1991) proposes a human genome diversity project (1991 October)
1992	NSF grant of $178,178 to Stanford University begins, on “Human Genome Diversity” (1992 May 15)
1992	Workshop 1 on Human Genome Diversity is held at Stanford University (1992 July 16–18); of an estimated 5000 populations on the basis of distinct languages, a sample of 400 populations and 25 individuals per population is proposed
1992	Workshop 2 on Human Genome Diversity is held at Pennsylvania State University (1992 October 29–31)
1993	Workshop 3 on Human Genome Diversity is held at the National Institutes of Health campus (1993 February 16–18)
1993	HGDP hearing is held before the Committee on Governmental Affairs, United States Senate (1993 April 26)
1993	Workshop 4 on Human Genome Diversity is held at Alghero, Italy (1993 September 9–12)
1993	WCIP meeting is attended by Hank Greely in Quetzaltenango, Guatemala (1993 December 6–10)
1994	MacArthur Foundation grant of $170,000 is given to Stanford University, “to support the work of the Human Genome Diversity Project's ethics committee and the development of a communications plan”
1996	First International Conference on DNA Sampling & Human Genetic Research: Ethical, Legal and Policy Aspects takes place in Montreal, with chapters by Greely (1997) and Cavalli-Sforza (1997) in the proceedings (Knoppers et al. 1997) (1996 September 6–8)
1997	A National Research Council report on human genome diversity viewed by the HGDP as conditionally favorable provides recommendations for how HGDP can proceed (Committee on Human Genome Diversity 1997), but news stories differ on its conclusions (McIlwain 1997; Pennisi 1997) and lead to correspondence and clarifications (Cavalli-Sforza et al. 1997; Schull 1997, 1998) (1997 October)
1997	The model ethical protocol is published (North American Regional Committee of the Human Genome Diversity Project 1997)
1998	Cann (1998) discusses the plan to maintain HGDP cell lines at CEPH (1998 June)
2002	Cann et al. (2002) report the availability of DNA from 1064 HGDP cell lines (2002 April 12)

Key sources include Greely (2001), Cavalli-Sforza (2005), Reardon (2005), and Stone and Lurquin (2005). Scientific findings after Cann et al. (2002) are discussed in the Appendix. Additional documentation appears in the Supplementary Information.

**Table 2.** Timeline of non-HGDP events that indirectly relate to the conversation.

Date	Event
1938	Luca Cavalli-Sforza enrolls at the University of Turin as a medical student, later transferring to the University of Pavia
1948	Cavalli-Sforza goes to Cambridge to study with R. A. Fisher
1958	The Nobel Prize in Physiology or Medicine is awarded to Joshua Lederberg, along with George Beadle and Edward Tatum
1970	Curt Stern retires from the University of California, Berkeley
1971	Cavalli-Sforza joins the faculty of the Stanford Department of Genetics, having been recruited by Joshua Lederberg; Marc Feldman also joins the Stanford faculty (in the Department of Biological Sciences)
1972	Richard Lewontin publishes “The apportionment of human diversity” (Lewontin 1972)
1974	Ruth Kirschstein becomes Director of the National Institute for General Medical Sciences (until 1993)
1976	John Moore's spleen is removed at the University of California, Los Angeles
1980	The paper of Botstein et al. (1980) leads to intense interest in restriction fragment length polymorphisms (RFLPs)
1980	The Nobel Prize in Physiology or Medicine is awarded to Jean Dausset, along with Baruj Benacerraf and George Snell
1984	Mary-Claire King begins working with the Abuelas de Plaza de Mayo in Argentina to use genetics to link disappeared children with their grandparents and other relatives
1987	Cann et al. (1987) study the most recent common ancestor of 147 mitochondrial genomes, the so-called “mitochondrial Eve”
1988	Judith Greenberg becomes Director of the Division of Genetics and Developmental Biology at the National Institute for General Medical Sciences (until 2015)
1990	Mary-Claire King's lab demonstrates existence of a gene for early-onset breast cancer (Hall et al. 1990)
1990	The California Supreme Court rules that John Moore does not have rights to profits from the tissues removed from his body
1991	Stanford holds a Centennial Symposium on the Human Genome Project (January 11–13)
1991	Bernardine Healy becomes Director of the National Institutes of Health (1991 April 9 to 1993 June 30)
1991	Allan Wilson (1934–1991) dies of leukemia (1991 July 21)
1993	Francis Collins becomes Director of the National Center for Human Genome Research, which later becomes the National Human Genome Research Institute (1993 April 4 until 2008 August 1)
1994	Cavalli-Sforza et al. (1994) publish *The History and Geography of Human Genes*
1994	The legislation known as the “Clinton health care plan” fails to pass the United States Congress
1994	Myriad Genetics applies for patent protection for discovery of breast cancer genes
1995	A patent is awarded to the Department of Health and Human Services based on a cell line from the Hagahai population
1997	Collins et al. (1997) propose a major study of human genetic variation distinct from the HGDP
1998	Craig Venter announces a plan to compete with the Human Genome Project in sequencing the human genome
2000	The draft sequence of the human genome is reported
2002	The International Haplotype Map Project is launched as the project descended from the paper of Collins et al. (1997)
2018	Luca Cavalli-Sforza (1922–2018) dies after having retired to Italy in 2008 (2018 August 31)
2022	The Nobel Prize in Physiology or Medicine is awarded to Svante Pääbo, referencing the HGDP Melanesians and Papuans in the prize description documents

Additional documentation appears in the Supplementary Information.

## The beginning of the HGDP

**Ramachandran:** Let's start with your involvement in the HGDP. The project begins with discussions among a small group of scientists, including Allan Wilson and Luca Cavalli-Sforza, in the late 1980s. We’d love to hear how you first became involved with the project that became the Human Genome Diversity Project.

**Feldman:** Luca was always interested in migration. He figured that when the Human Genome Project was going to be a reality, it would give a very good opportunity to study ancient migrations—to try to get as much data as he could from as many populations as he could, to study how the current population-genetic configuration of the world came about. As soon as it became obvious there was going to be a Human Genome Project, it was clear to Luca that that was missing a lot of the point—that you needed this other thing, too. Luca and I were meeting 3 to 4 nights a week for 35 years. He asked me to be involved because he thought that there would be great scope for modeling and data analysis when it eventuated.

**King:** It's a good guess that Luca had not thought about this idea for more than two minutes before he presented it to Marc.

In the late 1960s and early 1970s, I was a graduate student of Allan Wilson in Berkeley. My first introduction to genetics had been Curt Stern's course the last time he taught it, in 1967, before his retirement. I just loved it. As soon as he began speaking about population genetics, of course Luca's work was his focus. My dissertation project with Allan was evolutionary biology: the unexpected molecular similarity of human and chimpanzees.

By the mid-1980s, when I had my own lab in Berkeley, it was clear that restriction fragment length polymorphisms were going to revolutionize our capacity to work with human variation. I was still asking advice from Allan, and he suggested that I talk to Luca about coming to Stanford to learn “true population genetics.” So, I asked Luca if I could do an informal short sabbatical in his lab. He was extremely kind and said yes.

To Marc's point, he and Luca soon spoke with Allan about sampling people from all parts of the world. I wasn't present for their first conversations, but Allan and I talked a lot about the idea just after. He thought it was good but wondered about the design.

**Greely:** I was on the planning committee for a 3-day symposium Stanford put on about the then-new Human Genome Project. It was January of ‘91. The planning committee was chaired by Paul Berg, and it included Lucy Shapiro and David Botstein. They wanted a law professor. They told my dean, and since I was the only one at the law school who knew how to spell DNA, I was volunteered for it. I loved the planning committee. It was so much fun to see both Paul and especially Lucy constantly teasing David, and David's responses. I just enjoyed the people.

Until the Clinton Health Plan crashed and burned in ‘94, I still thought I was a health policy person. We ended up deciding that I would give a talk on health insurance and the implications of genetics for health insurance.

The talk was well received. It actually ultimately became a chapter in the Lee Hood/Dan Kevles *Code of Codes* book [Kevles and Hood (1992)]. Luca was introducing everybody on that panel, and he introduced me. I can't remember now the Latinate term he had for it, but as he was reading where I've been and gone, which really wasn't that many places, he decided I must have the genetic trait of nomadism, as part of his humorous introduction.

Well, I enjoyed the talk. In the summer of ‘92 Marc and Luca invited me to lunch. It was at the Faculty Club, to ask some questions about this project they were doing. I said I thought it sounded really interesting, and that is when I first got pulled in.

**Rosenberg:** Let's talk about the early arguments for why it was important to do a genome diversity project, and how these thoughts coalesced in an article in *Genomics* in 1991 [Cavalli-Sforza et al. (1991)].

**King:** Because we are not one genome! The evolutionary perspective on all of life requires that we recognize extraordinary diversity and the forces of evolution that are behind it.

My recollection is that Victor McKusick invited us to write a commentary for *Genomics* on this idea of a worldwide human variation project, with Luca and Allan as authors. They had not written together previously, and you can't imagine two more different people as the major contributors to our field. They were both progressive—but differently so. Allan was very left-wing, grew up on a New Zealand farm, and maintained a Bohemian kind of lifestyle his entire life. Luca, of course, had a very different background: urban and European. They were never close friends, but had huge respect for each other.

My goal was to help Allan and Luca to work together on this commentary. Also part of the story was Charles Cantor at the Department of Energy, which had a lot of capacity for sequencing. Charles was very interested in the project from the technical point of view: could one actually apply sequencing to the kinds of numbers of samples that Luca envisioned? It seemed important to bring DOE sequencing capacity to bear, and Charles was completely open to that. Another interested person was bioethicist Bob Cook-Deegan. The idea was to put some ideas down on paper so that the new genomics community would recognize its importance.

**Rosenberg:** We would like to know more about the interactions between Allan and Luca. In the lore of the project, there's a fair amount of weight placed on the somewhat competing visions of how to do the sampling, whether population-based sampling in Luca's view or a more geographic-based sampling in Allan's.

**King:** Allan thought in terms of sequence, which meant that every individual offered a data set, with the metric being numbers of DNA base pair differences between individuals. This was a different perspective than calculating allele frequencies and comparing frequencies between population clusters. Both perspectives are legitimate, but different.

Luca and Marc were thinking about sampling strategies. Allan felt strongly that the best way to sample was to represent the geography of the planet, so to sample either one or a very small number of people from as many different geographic regions as possible, always sampling people who had a depth of ancestry in that region. Luca thought of clusters of people defined by language groups, because he was thinking of allele frequencies. Allan was thinking of sequence.

**Rosenberg:** That's an interesting tension in the field that continues into the 21st century.

Looking back 35 years at what was most significant in human population genetics at the time, there's Mitochondrial Eve from Allan's group [Cann et al. (1987)], with this individual sequence-based perspective, and then there's Luca's more population-based book on history and geography of human genes [Cavalli-Sforza et al. (1994)].

**Feldman:** I think it's really important to emphasize that Luca was very influenced by linguistics. We would sit there with the Ethnologue, that big volume [Grimes (1992)]. And that's where this number 5,000 populations that you see in the first report of the project came from, because there were approximately 5,000 languages in the Ethnologue at that time.

**King:** Allan realized that he was ill only seven months before he died [of leukemia in July 1991]. He was thinking about this project all the time. It's what we talked about in my last visit with him. With his death, his “grid sampling” idea faded in the presence of the strong, coherent vision of Luca and Marc. Most of all, I think everyone felt that the main thing was to get it going…

**Feldman:** And also to utilize the resources that were already available that had been collected. I wanted to mention one other thing that was relevant to Luca's beginning there: the paper that [Richard] Lewontin did in 1972. Luca loved that paper, the one on apportionment of human diversity [Lewontin (1972)]. Luca was an analysis-of-variance person. That really was his cultural legacy from his time with [Sir Ronald] Fisher.

## The planning workshops

**Rosenberg:** Let's talk about what happened after the 1991 *Genomics* article. The article attracted some attention, including a news story in *Science* [Roberts (1991)] and a letter to the editor from Mark Weiss, a program officer at the National Science Foundation [Weiss (1991)]. Mark Weiss's interest led to a proposal to NSF to run a series of workshops to develop the idea for a genome diversity project into a more concrete scientific project, and this proposal is funded for $178,178. We are wondering if you have any specific recollections from the workshops in 1992 and 1993 to develop the project.

**Feldman:** My recollection is at the first meeting at Stanford, we spent a huge amount of time talking about statistics and about sampling sizes. And I remember walking down what is now the pathway near Bytes Cafe, with [Joe] Felsenstein and Luca, and really going hammer and tongs on how many people you would need to sample.

At the second meeting, we talked about trying to write a proposal that would involve actually getting the samples. And the need to get people like Hank involved. How consents might be incorporated from tribes or peoples that had not had that sort of thing historically. Where the samples could go… people got down to the nitty gritty.

**King:** My memory is that the idea of community consent was real from the very beginning.

**Greely:** The third meeting was probably the first time I met anybody from NIH. Memories are flooding back, I’ve eaten the little madeleine. It was a really interesting and intense day, and I left thinking, “This is even more complicated than I thought it was going to be.”

**Rosenberg:** The project is estimating that it hopes to obtain $25 million, and there are program officers from NSF, NIH, and DOE at the meetings. We’d like to understand how the future funding plan for HGDP was developing.

**Feldman:** Irene Eckstrand from NIGMS [National Institute for General Medical Sciences] was really very nice. She was a card-carrying population geneticist, and when she became a program officer for genetics, a lot of what she saw was population genetics. I ended up chairing the genetics study section and had a lot to do with Irene, who was so sympathetic, and tried to move up the narrative and the conversation to Judith Greenberg [Director of NIGMS Division of Genetics and Developmental Biology] and the bosses.

**King:** Ruth Kirschstein [Director of NIGMS] was incredibly supportive of me and of my breast cancer work. She listened to her investigators and to her staff, young or established, and regardless of fields. When she heard about the HGDP from Irene, even though it wasn't her field at all, she believed in Irene's view of it.

**Feldman**: Mark Weiss at NSF was sympathetic, but NSF had pennies. NIH would have to be involved somehow.

## A Senate hearing

**Rosenberg:** We’d like to talk about an interesting moment in the early development of the project, a hearing that took place at the US Senate Committee on Governmental Affairs on April 26, 1993. By this point, three planning workshops had already taken place, using the NSF funding. The most recent one was held at NIH in February of 1993 and had a significant presence of program officers, and a lot of discussion of ethical challenges such as sample collection, cell line management, how DNA would get distributed, databasing, and human subjects protections.

The Senate hearing was one of the first significant moments where the project was having a public conversation, beyond the scientific community. Luca and Mary-Claire represented the project at the hearing, and Francis Collins, who had just been appointed Director of the National Center for Human Genome Research (NCHGR), later the National Human Genome Research Institute (NHGRI), also testified.

Mary-Claire, your testimony spoke to the importance of the project, both the human evolution and biomedical dimensions, and you also mentioned your work using genetics to connect disappeared children in Argentina to their grandmothers, the Abuelas de Plaza de Mayo. Tell us about preparing for the hearing.

**King:** I felt that my assignment was to humanize the project and to link it to medical research; to clarify that we were not an island off in a sea of theory, and that human diversity was a good use of the new technology, just as medical research was. Judith Greenberg said to me (I'm paraphrasing here): you need to get this right… make sure that the connections between the Human Genome Diversity Project and Human Genome Project and biomedical research all make sense. I took her advice seriously.

**Rosenberg:** In retrospect, this hearing also appears to be the first moment when it looks like HGDP will have difficulty with funding from NIH. Is that your sense, looking at the transcript now?

**Feldman:** [examining the transcript] I think it's interesting, [Francis] Collins in his oral testimony raises more doubts about the informed consent than in his written testimony submitted in advance, and in particular, “will the Third World feel exploited?”

The points that he makes in person raise the difficulties that we'd already discussed. If you're going to sell something to the Senate, then you might not raise all the difficulties that the people who are doing the project already raised.

**Greely:** He was clearly *for* the Human Genome Project. That was the baby he was trying to protect. This was, as he notes, his first Congressional hearing as Director. He had just become Director a couple of weeks earlier in April.

During the Q&A in this transcript, you've got Collins saying he supports the Diversity Project, there are really interesting goals in common, but “it would be difficult for the Genome Project to expand its umbrella to take on other more diverse efforts, like the Diversity Project, without expanding its funding.” So he was already clearly distancing.

**King:** Bear in mind levels of funding at NIH in that year. This was early in the Clinton administration. NIH funding was going nowhere but up.

**Ramachandran:** Mary-Claire, at this time, you're in an incredible period of professional success after your major finding of linkage to an early-onset breast cancer gene in 1990 [Hall et al. (1990)]. It's interesting to us that of all things you could be doing in human genetics at this time—you’re working on identifying the breast cancer gene to which you had found linkage, your work on genetic identification of missing children is getting international attention, you’d been considered by NIH Director Bernardine Healy to direct the National Center for Human Genome Research and the Human Genome Project—you’re at this Senate hearing devoting a large part of your energy to HGDP. What was drawing you to HGDP?

**King:** It was the way to integrate evolution with human genetics, using technology that was coming online very, very fast. It seemed to me that biomedical questions and human diversity and evolution questions were absolutely integrable. We were saying, in effect, that people from populations that you all have never visited are just as important to understanding health and disease as the people whose DNA will be the first [human genome] sequence.

## Supplementary Material

iyaf273_Supplementary_Data

## Figures and Tables

**Table 3.** National Science Foundation grants related to the Human Genome Diversity Project, totaling $887,701.

Title	Principal investigator	Institution	Co-PI	Dates	Amount
Human Genome Diversity*^a^*	Marcus Feldman	The Leland Stanford Junior University	L. Luca Cavalli-Sforza, Kenneth Weiss, Mary-Claire King, Kenneth Kidd	05/15/1992–04/30/1997	$178,178
Pilot HGDP: Post-Collection and International Ethical and Legal Issues in the Proposed Human Genome Diversity Project	Henry Greely	The Leland Stanford Junior University		04/15/1997–12/27/1999	$36,000
Pilot HGDP: Cross-Cultural Issues in Genetic Research with Plains Apaches: A Pilot Study for the Human Genome Diversity Project	Thomas Carter	University of Oklahoma Health Sciences Center	Morris Foster	04/15/1997–03/31/2000	$110,000
Pilot HGDP: Banking, Sequencing, and Haplotyping of Archival Human DNA Samples	Kenneth Weiss	Pennsylvania State University	Deborah Nickerson, Andrew Clark	04/15/1997–03/31/2000	$81,400
Pilot HGDP: Developing Genetic Markers Informative in all Populations	Pui-Yan Kwok	Washington University School of Medicine		04/15/1997–03/31/2000	$107,685
Pilot HGDP: Efficient Recovery of Useful Markers: Application for use in the Human Genome Diversity Project	Glenys Thomson	University of California, Berkeley		05/01/1997–10/31/1999	$124,123
Pilot HGDP: Assessment of High-Throughput Assays for the PCR based Genotyping of Classical Polymorphic Systems*^b^*	Mark Shriver	Allegheny University of the Health Sciences	Robert Ferrell, Ranjan Deka	05/01/1997–04/30/2000	$87,598
Pilot HGDP: Contribution of Alu repeats to Human Genomic Diversity	Mark Batzer	Louisiana State University Health Sciences Center		09/01/1997–08/31/1999	$54,999
Pilot HGDP: Optimization of DNA Analysis in Nontraditional Settings	Lynn Jorde	University of Utah	Michael Bamshad	09/15/1997–02/28/2001	$107,718

In addition, a grant for $170,000 from the MacArthur Foundation was given to Stanford University in 1994 “*To support the work of the Human Genome Diversity Project's ethics committee and the development of a communications plan*.”

^a^$38,000 of this grant was transferred to NSF from the National Center for Human Genome Research, the predecessor of the National Human Genome Research Institute.

^b^A portion of this grant was transferred when the PI moved institutions.

**Table 4.** Original questions that the report of the first HGDP workshop proposed in 1992 that the project would address.

Number	Question(s)	Three notable HGDP-CEPH studies whose analyses relate to the question
1	What are the frequencies of various genes in different populations?	Rosenberg et al. (2003b), Li et al. (2008), and Pickrell et al. (2009)
2	What are the heterozygosities or diversity within and among populations?	Prugnolle et al. (2005), Ramachandran et al. (2005), and DeGiorgio and Rosenberg (2009)
3	What special phenotypes, such as diseases, characterize certain populations, and are these correlated with genes for genotypes?	Myles et al. (2008), Ding and Kullo (2011), and Berg and Coop (2014)
4	How do within- and among-population statistics compare? What is the variation among various human populations?	Li et al. (2008), Rosenberg (2011), and Liu et al. (2023)
5	What is the history of human population size? Were there bottlenecks or sudden expansions?	Ramachandran et al. (2005), Liu et al. (2006), and DeGiorgio et al. (2009)
6	What is the history of population subdivision?	Rosenberg et al. (2005), Pickrell and Pritchard (2012), and Grundler et al. (2025)
7	What is the relative importance of random drift and selection? [What is] the importance of selection for structural or regulatory genes?	Ramachandran et al. (2005), Hofer et al. (2009), and Carja et al. (2017)
8	What is the evolutionary relationship of humans to other primates?	Green et al. (2010), Reich et al. (2010), and Bergström et al. (2020)
9	Is there spatial patterning among populations? Are populations that are geographically close together more closely related? What are the correlations of genotype and distance? Are there geographical clines, and if so, why?	Rosenberg et al. (2002), Lawson Handley et al. (2007), and Coop et al. (2009)
10	How is disease susceptibility distributed in and among populations? What accounts for important genotype–phenotype relationships and for genotype-by-environment interactions within and among populations?	Myles et al. (2008), Berg and Coop (2014), and Blair and Feldman (2015)
11	What are the relationships among genetic, cultural, linguistic, and ecological variables?	Hancock et al. (2008), Coop et al. (2010), and Creanza et al. (2015)
12	How do mutation and recombination rates vary among populations and among loci?	Conrad et al. (2006), Pemberton et al. (2009), and Bergström et al. (2020)
13	How much linkage disequilibrium is there in the population, and is it caused by stratification or by epistasis?	Jakobsson et al. (2008), Li et al. (2008), and Bosch et al. (2009)

We include in question 8 ancient genomics analyses with Neanderthal and Denisova samples.

## Data Availability

The study contains no data. Explanatory notes appear in supplemental material. Supplemental material is available at GENETICS online.

## Interactions of HGDP with indigenous populations and activists

**Ramachandran:** Let's talk about the HGDP and indigenous groups. Is there anyone who was particularly memorable in trying to build relationships with indigenous populations?

**Greely:** John Moore was an outsized character, both physically and every other respect. He was an anthropologist and expert on the Cheyenne. He had a book about the Cheyenne that I bought and read. When he started with us, he was at the University of Oklahoma, but he shortly thereafter moved to the University of Florida. He was a big personality, quite a storyteller, and he was a fascinating guy to listen to. He hosted a meeting on the HGDP at Florida at some point [and] had very good connections with some of the tribal nations.

Catherine Twinn was on the North American committee of HGDP and very supportive. Her husband was the leader of the [Sawridge] Cree of Slave Lake, Alberta, which was one of the most economically successful First Nations in Canada. I hear from her every 5 years or so. Just a lovely person. We went to a meeting of indigenous peoples in Wisconsin, it was the day when, while we were driving to this place way out in the mosquito-laden lakes of Wisconsin, the slow-motion helicopter followed O. J. Simpson.

**Rosenberg:** Let's discuss the criticisms from activists that arose in spite of these relationships that the project was building. Tell us how the public criticism of the project emerged, what it entailed, and what the initial reactions were within the project.

**Greely:** I don't remember how RAFI got involved, Rural Advancement Foundation International. I do think this is one of those sad, sad stories. At the Penn State meeting that I wasn't at, in October 1992, there was discussion about, how are we going to pick what populations we should sample. The report of that meeting had the reasonable idea that you should make sure that we sample the ones that are disappearing, and called them “isolates of historical interest,” IHIs. The acronym ended up getting used, and RAFI picked that up, and said [paraphrasing] “we are not merely of historical interest. We are alive and kicking. This is bioimperialism, biocolonialism,” et cetera.

And of course none of that had been intended, but it got played that way. And RAFI started a form of criticism. They got to the World Council of Indigenous Peoples, which did invite us to come down to their meeting in Quetzaltenango, Guatemala, that was December of “93. The same time, we're beginning to have some interest from ethics groups: the HUGO [Human Genome Organisation] ethics committee, the UNESCO [United Nations Educational, Scientific and Cultural Organization] ethics committee. It's becoming a topic of discussion among people who are doing ELSI work.

**Rosenberg:** Tell us about the Guatemala meeting.

**Greely:** The WCIP, the World Council of Indigenous Peoples, was heavily oriented toward the Andean Cordillera. So there were lots of representatives from Colombia, Peru, Ecuador, and Bolivia. Ken Weiss, who had done work in Central America and was fluent in Spanish, he and I were supposed to go there together, and just before we're supposed to go, he canceled. So I ended up flying to Guatemala City alone, renting a car, driving to Quetzaltenango with basically no Spanish, being accused of being a CIA agent. But you know, somebody ultimately said “well, you're really very pleasant for a CIA agent.” It was a daunting experience. The WCIP decided we were the “vampire project” and evil, despite my disclaiming the CIA role.

**Rosenberg:** The patent environment of the time seems important. It seems like the 1990s is a time of heightened concern about patenting biological material. Some populations are worried about commercial exploitation of their DNA. The Human Genome Project has a lot of patent worries. There's the Myriad patent on BRCA1 in that period.

**Greely:** If you asked people about geneticists exploiting people financially, John Moore's spleen was the first thing that came up. This is a different John Moore, I interviewed him.

John Moore was a guy who was working on the Trans-Alaskan pipeline system. He got sick. He was lucky enough to have a doctor in Alaska who recognized that he had an unusual leukemia called hairy cell leukemia.

His dad was a physician in the San Gabriel Valley, and his dad did some research on good clinicians for his son. He said, son, there are three people in the world who are the world's experts on hairy cell leukemia. One's in London, one's in Chicago, and one's here at UCLA.

So he went to UCLA, where he was treated by Dr. David Golde, who was very interested and was publishing on hairy cell leukemia. What kind of cell becomes malignant in hairy cell leukemia? They didn't know. So he's doing research on the cell types. He's published something saying, they're B cells, which they are.

He does the research on Moore. The canonical story has him taking the spleen out, although he's an oncologist and not a surgeon. The best evidence is that the spleen weighed about 6 kg, although both Moore and Golde remembered it being closer to 20 pounds.

So they weren't taking the spleen out in order to do research on it. They were taking it out, because if that sucker popped—a normal spleen should be less than about a half pound—so if that pops and spills into the peritoneal cavity, bad stuff happens.

Dr. Golde gets a cell line going. UCLA gets a patent. Golde sends the cell line to Robert Gallo, who's big on viral-induced tumors. He discovers the world's second known human retrovirus in this cell line, HTLV-2. He later tries to name the third known human retrovirus HTLV-3, but it ended up getting named HIV instead. The cell line becomes a big deal, and the National Cancer Institute sends people to research Moore and his family. Golde takes a lot more biological samples from Moore, who wonders what's going on. Golde tries to get him to sign another consent. Moore gets suspicious, calls a lawyer, brings a lawsuit. The California Supreme Court in 1990 says, no, those cell lines are not your property.

That is the first big “who owns your genes, who owns your cells,” story. The canonical version of it is all about “bioscientists are stealing from people.” So you begin to see “bioimperialism” come up.

**Rosenberg:** We see references to several incidents involving patents and cell lines in the 1990s. There was one incident about New Guinea?

**Greely:** The Hagahai, a New Guinean tribe. That's the one that really pissed me off because I had long conversations with people at RAFI about how we had nothing to do with it. We knew nothing about it, and, in fact, the poor American anthropologist, she'd gotten NIH to patent the cell line derived from the Hagahai because it had a variant of the HTLV-1 virus. In order to protect any rights the Hagahai had, she wanted to make sure that NIH would have a patent—so if it ever got used, NIH had agreed to give the royalties to the Hagahai. Well, she got hung out to dry as a bioimperialist.

I explained the HGDP had nothing to do with it, and RAFI still put out a press release that said, you know, the “vampire project” strikes again, blaming us for the Hagahai situation.

**Rosenberg:** We're wondering about any reflections during the time of the criticism from the activists, on the issue of who represents indigenous populations in regard to participation in research.

**Greely:** One thing that made it relatively easy for the HGDP North American Committee was the indigenous peoples we were mainly concerned about were native peoples of the Americas, and in both the United States and Canada, there were recognized governments for most, but not all of those. So one position we took was, tribal government is a necessary part of the group consent.

So it's like a state. And so you'd have to get their approval. But we also said that there will be situations where there may be traditional leadership groups. And I think this really came from [anthropologist] John Moore, groups of elders or others who are not officially the government, but are recognized within the population as leaders, and in that case you would need to get their consent as well.

I actually liked a lot of the indigenous people I talked to who were strongly opposed to the project. The attacks from indigenous peoples, from people who think they represent indigenous peoples, I was fine with, as long as I thought they were in good faith, which I didn't think about RAFI. But then there was the NIH. It was the undercutting from within science—that was the second front. So we were attacked on two fronts, and one of them was understandable, and one of them was just plain frustrating and never exactly overt.

## Hosting the cell lines at CEPH

**Rosenberg:** At some point, in 1996, Jean Dausset, the Nobel laureate for his work on HLA [Human Leukocyte Antigen], who is located in Paris at CEPH, the Centre d’Etude du Polymorphisme Humain, expresses an interest in hosting HGDP cell lines. A plan to host cell lines at CEPH is clear by the time of a 1998 article by Howard Cann from CEPH [Cann (1998)], and Howard leads the 2002 *Science* article announcing the availability of DNA from the cell lines [Cann et al. (2002)]. Do you know why CEPH came to be the host of choice for the cell lines?

**King:** In order to enable matching by HLA type for organ transplants, Dr. Dausset organized an international collaboration for HLA genetics and tissue typing. This collaboration was truly international, with labs from *everywhere*. Every lab was given the same panel of antigens, with contributions of fresh blood from all parts of the world, white cells processed in Paris, and antigens distributed to laboratories worldwide. As patients everywhere needed organ transplants, and as organs became available, it was the responsibility of the local lab to type the white cells of the donor or the recipient with the antigens of this panel and report back. A roster of results was kept in Paris.

Everybody met every year or so to discuss how this worked. The meetings were attended by technicians, transplant physicians, patients and their families. Dr. Dausset presided in a very gentle way, and was extremely good at pulling together people across continents, across social class, across professions with a common goal.

So, it made sense that he would take on hosting the HGDP. He was not afraid of complexity, either scientific or logistical.

**Feldman:** Howard Cann had been professor of pediatrics and genetics at Stanford, and he ran the chromosome testing lab. Howard had been a faculty member at Stanford and then moved to CEPH. So he was a link between Stanford and Paris. He became quite important to the success of putting all of the cell lines together.

## Working with Luca

**Ramachandran:** One of the really impressive things about the project is how hard everybody worked on it, given all the challenges, and despite no significant funding for so long, and the criticism that you were fielding. What made it so important to keep persisting?

**Greely:** Not letting the team down. We were committed to the ideas, but also to each other.

**King:** It didn't feel like working hard. We had a charming leader.

**Ramachandran:** It does seem like everybody working together on HGDP in the early years really enjoyed working together.

**King:** No question. I mean, who wouldn’t? Going to one of these meetings, we would have conversations about history and linguistics and anthropology and geography and genetics and evolution and migration. It was like opening a gift box from nature.

**Greely:** It was an entrée into a bunch of different worlds. I've always had academic attention deficit and hyperactivity disorder. To be around smart people who know a whole bunch of things that I don't know anything about? That was heaven.

**Feldman:** People were united in their respect for Luca. Because if there had ever been a Renaissance man, it was Luca. My wife and I went to Japan with him. We got a guide, and he took us into remote parts of Japan… Luca knew much more than the guide. We took a trip with him to Taiwan, same thing happened. I was unbelievably privileged to be around him.

**Greely:** And always so charming, and such a great personal presence, and an ability to make you feel at ease, just one of the most remarkable people I've ever met.

**King:** Luca had the capacity to see the best in people. I think you could drop Luca anywhere on the planet, and he would soon make friends.

**Greely:** Even though he was clearly a man from a different century almost. Such a different background and culture, so dated in some ways that I think, for some people who didn't get to know him, was off-putting… he was a person from Italy before World War II.

**Feldman:** It's interesting how he dropped out of school. He hated high school, and he studied at home. Then he got admitted to university. But he knew more coming out of high school than most undergraduates.

**King:** I heard stories from his time as a medical student. It was in the middle of the war, and you could be picked up by “recruiters” and forced into the army. It was more dangerous after Mussolini was ousted, because the Germans took over the country. Students stayed in different houses on different nights. The recruiters did not come directly into the medical schools (because medical students were legally exempt), so they could still attend class, but they would arrive by riding their bicycles on off-roads so that they wouldn't be caught on the way to or from the university.

**Feldman:** He hated the practice of medicine. After he finished in medical school, he was in a hospital for a little while. He hated it, and that's why he really preferred doing bacterial genetics. He was really a brilliant bacterial geneticist, I think he should have shared the Nobel Prize with [Joshua] Lederberg for sex in bacteria.

The thing one has to understand about Luca's modus operandi is that he never wanted to work on what other people were working on. If you look at the sequence of stuff that he did, he initiated so many different things that other people started to learn about.

**Greely:** It's interesting, you want to talk about the history of the HGDP, and instead, we keep talking about Luca. That says something about what held the HGDP together.

**Rosenberg:** We remember Luca as a very optimistic, forward-looking person. How did he experience the criticism of the HGDP?

**Feldman:** He never dwelled on it. I think one of the things that I find interesting in retrospect is we were more fazed by this opposition than Luca was.

**Greely:** At the meeting about DNA sampling held in Montreal, in ‘96, there was a demonstration against the project. Well, the conference provided a bodyguard for Luca, a middle-aged Quebecois, he'd been a professional boxer. Luca thought this was very funny.

**King:** It was very helpful to me to be around someone so capable of rising above a vicious personal attack. You learn from people like that.

**Greely:** He did take the bodyguard though.

**Rosenberg:** What do you think was Luca's favorite work to emerge from the HGDP?

**Feldman:** He was always interested in migration. You can see that from his theoretical work on gene surfing. He was always drawn back to Fisher's work, his work on Neolithic spread of farming [Ammerman and Cavalli-Sforza (1979)], he phrased it in terms of Fisher's wave of advance from the 1937 paper [Fisher (1937)]. The big book on human genetics is really a book about migration of humans. After he switched from bacterial genetics to asking questions about humans, he did his first data collection on the parish records in Italy. That work continued until the Princeton monograph on consanguinity [Cavalli-Sforza et al. (2004)]. He was also a great admirer of the Kimura-Weiss papers on stepping-stone migration [Kimura and Weiss (1964); Weiss and Kimura (1965)]. He liked our 2002 paper [Rosenberg et al. (2002)], he expressed admiration for it. Thinking about Luca's predilection with migration, I think Sohini's paper on migration [Ramachandran et al. (2005)]—the rate of decline of heterozygosity out of Africa to the Americas—I think that for Luca, that was the most delicious finding.

## Coda

**Rosenberg:** In an obituary for Luca in the *Stanford Report*, Jonathan Pritchard said, “HGDP was a huge contribution. It was really one of the first projects to create a comprehensive view of worldwide genetic diversity. Since then many large international projects have built on that idea recapitulating aspects of HGDP. But the fundamental idea came from Luca.”

Because the HGDP-CEPH panel had so many more populations than, for example, the HapMap Project, the HGDP-CEPH panel has had the function of providing data that can be used to test hypotheses about the value of diversity in genomic data sets. Lately, the human genomics field has moved toward advocating for diversity in genomic samples. However, the connection is not always made that the HGDP was early to this idea. What are your thoughts about this aspect of the HGDP's legacy?

**Greely:** Ultimately, what's important is the field is coming around to the positions that we think are good positions. Whether HGDP gets credit for it or not doesn’t matter. It’d be nice, but it doesn’t matter.

**King:** The best possible credit is to be so taken for granted you're not even mentioned any more. In the long run, diversity will be at the basis of the way we think about people. A respect for populations of all ancestries was what defined the Human Genome Diversity Project.

**Greely:** And causation is almost always unknowable. Did HGDP contribute to the current greater recognition of these issues? I think it did. Can I prove that? No. But can I take pride in what we did that at least pointed in the right direction? Yeah.

**Rosenberg:** You’ve all had many successes throughout your careers. This project occupied a lot of everyone's energy over a significant period. How do you think about HGDP in the totality of the work you've done?

**Greely:** It set my career. I’d found my field. And what a field it turned out to be, I call it “law and biosciences,” but it's the intersection of society and bioscience. And I've worked on genetics, on neuroscience, on stem-cell research, on assisted reproduction, on a whole bunch of different stuff that isn’t necessarily connected. But what started it was the HGDP. The HGDP had success, but was not as successful as we had hoped. But for me personally, it did really determine the rest of my career, for which I'm very grateful.

**King:** It solidified for me the importance of incorporating evolutionary thinking into biomedical research.

**Feldman:** For me, it was a continuation of things that I’d been involved with with Luca for years. Among the reasons why it remains a very high point is because of you guys [to Noah and Sohini]. It really enabled students to make significant contributions. I mean, it was so fruitful. So fruitful.

I have a paper that we're about to submit on parallels between genetic variation and linguistic variation. Whenever you talk about that kind of thing, you’re thinking about the things that Luca was thinking about 40 years ago.

**Ramachandran:** What do you wish researchers entering the field today knew about the project?

**Feldman:** I really think you have to look at it as a part of a tradition that probably began with Lewontin (1972), Cavalli's papers with Bowcock [Bowcock et al. (1991, 1994)] and Barbujani [Barbujani et al. (1997)], then our papers [Rosenberg et al. (2002, 2005); Ramachandran et al. (2005); Li et al. (2008)]. I think it's a straight line through. And the conclusions about within-population versus between-population apportionment of human diversity just keep getting stronger. It seems to me to be important to teach that historical continuity.

**Greely:** And I'd like them to know about the ethical and political difficulties we faced and the progress we made trying to—not solve them, these are problems that never get solved—but to manage them in a better way. Mainly involving respect for the people whose heritage you’re looking at.

**King:** It was a good project. We did it well. We had a great time, and the cell lines and the data and the science are still there.

## Appendix. Partial bibliography of the scientific contributions of HGDP

The first HGDP workshop report in 1992 had listed 13 specific questions for the proposed research agenda of the HGDP, a sort of “Hilbert problems” list for the field of human population genetics. Table 4 lists these questions, along with notable studies that, from 2002 onward, addressed them with data from the HGDP-CEPH panel.

More generally, since the announcement by Cann et al. (2002) of the assembly of the HGDP-CEPH cell lines, applications of the lines and associated genetic data have been numerous. Cavalli-Sforza (2005) summarized the early contributions (2002–2005), and Aneli et al. (2022) provided a more recent retrospective. Applications of the HGDP-CEPH panel often cite one or more of the main studies that included, as a substantial component, the dissemination of genetic resources focused on the panel (Cann et al. 2002; Rosenberg et al. 2002, 2005; Ramachandran et al. 2005; Rosenberg 2006; Jakobsson et al. 2008; Li et al. 2008; Pickrell et al. 2009; Bergström et al. 2020). HGDP-CEPH genotypes and sequences have also been gathered in subsequent resources that aggregated data from multiple sources (Xing et al. 2010; Pemberton et al. 2013; Mallick et al. 2016; Koenig et al. 2024). Because studies that have made use of the aggregated resources have not necessarily referred to the original sources that were combined, references to the HGDP-CEPH panel itself are often absent in applications that rest on it; the HGDP-CEPH panel has, in effect, been stitched into the fabric of the field of human population genetics; in Mary-Claire's phrase, it is “so taken for granted” that it is “not even mentioned any more.”

Of the thousands of scientific articles that have relied directly on the HGDP-CEPH panel, many fall into the following six broad areas. Our bibliography of contributions based on the HGDP-CEPH panel is far from exhaustive; we focus here on the use of the panel for scientific advances in the spirit of the research fields that produced it, centered around human population genetics, and also including adjacent areas of human evolution, biomedically oriented human genetics, and the broader science of population genetics.

**1. Human evolution, population-genetic structure, and demographic processes.** Data based on the HGDP-CEPH panel have been prominently used for analyses of human evolutionary history, population structure, and the geographic distribution of genetic variation. Topics of genetic diversity, the partition of genetic variation, population structure, and bottlenecks, migrations, and range expansions have been a focus, both for many of the studies most directly associated with the panel (Rosenberg et al. 2002, 2005; Ramachandran et al. 2005; Jakobsson et al. 2008; Li et al. 2008; Bergström et al. 2020), as well as for many others that use it (Excoffier and Hamilton 2003; Rosenberg et al. 2003a; Zhivotovsky et al. 2003; Serre and Pääbo 2004; Liu et al. 2006; DeGiorgio et al. 2009; Ramachandran and Rosenberg 2011; Lawson et al. 2012; Wang et al. 2012; Jay et al. 2013; Hellenthal et al. 2014 ; Hunley et al. 2016), including several notable recent articles (Wohns et al. 2022; Hu et al. 2023; Cousins et al. 2025; Grundler et al. 2025).

HGDP-CEPH genome-wide data have been used to analyze signatures in features that vary spatially along the genome, including haplotype and linkage disequilibrium patterns, runs of homozygosity, and identity-by-descent sharing (Jakobsson et al. 2008; Li et al. 2008; Bosch et al. 2009; Kirin et al. 2010; Leutenegger et al. 2011; Henn et al. 2012; Lawson et al. 2012; Pemberton et al. 2012; Szpiech et al. 2013; Pemberton and Rosenberg 2014; Prates et al. 2023). Studies using the HGDP-CEPH panel rode a wave of evaluations of the phenomenon of “allele surfing,” where an allele rises to a high frequency at the leading edge of an expanding population, examining the population-genetic effects of expansions that take place through sequential founder effects (Ramachandran et al. 2005; DeGiorgio et al. 2009, 2011; Hofer et al. 2009). Some of the contributions from studies of population structure and demographic history appear in the conversation with Marc, Hank, and Mary-Claire, including updates to Lewontin's apportionment of human diversity (Rosenberg et al. 2002; Li et al. 2008), and Marc's comment of the “delicious finding” of the decline of heterozygosity outward from Africa (Ramachandran et al. 2005; see also Prugnolle et al. 2005).

**2. Ancestry for specific samples.** HGDP-CEPH data often serve as a backdrop for population-genetic studies of new samples. Studies have combined HGDP-CEPH data with populations from around the world, including African populations (Tishkoff et al. 2009; Henn et al. 2011; Schlebusch et al. 2012), Native Americans (Wang et al. 2007; Reich et al. 2012; Verdu et al. 2017), populations from India (Rosenberg et al. 2006; Moorjani et al. 2013; Basu et al. 2016; Kerdoncuff et al. 2025), Australasians and Pacific Islanders (Friedlaender et al. 2008; Rasmussen et al. 2011; Bergström et al. 2017), and admixed groups of the Americas (Wang et al. 2008; Bryc et al. 2010; Moreno-Estrada et al. 2013).

One form of study in this class has used the HGDP-CEPH panel to assist in understanding genetic ancestry in biomedical samples (Xu et al. 2009; Zakharia et al. 2009; Manichaikul et al. 2012; Thornton and Bermejo 2014; Berardinelli et al. 2022; Wendt et al. 2023); another uses it to assess relationships of ancient samples to modern populations (Green et al. 2010; Raghavan et al. 2015; Sümer et al. 2025). Aneli et al. (2022) noted the importance of an HGDP-CEPH Papuan individual in an early assessment of the relationship of the original Denisova Cave ancient sample to extant human populations (Reich et al. 2010); indeed, the HGDP-CEPH Melanesian and Papuan data of Li et al. (2008) were mentioned in the description of the Nobel Prize later awarded to Svante Pääbo for his work with ancient DNA.

**3. Geographic patterns in different types of loci and at specific loci.** The HGDP-CEPH panel has been used to study genotypes and geographic patterns in many types of loci. In addition to microsatellites (Rosenberg et al. 2002, 2005; Ramachandran et al. 2005) and single-nucleotide polymorphisms (Jakobsson et al. 2008; Li et al. 2008; López Herráez et al. 2009; Patterson et al. 2012), examples include Alu insertions (Wildschutte et al. 2015), forensic loci (Santos 2015; Algee-Hewitt et al. 2016), KIR immunologic loci (Hollenbach et al. 2012), LINE-1 retrotransposons (Beck et al. 2010), nonsense SNPs (Yngvadottir et al. 2009), short insertion-deletion polymorphisms (Rosenberg et al. 2005; Bastos-Rodrigues et al. 2006), and structural variants (Jakobsson et al. 2008; Itsara et al. 2009; Almarri et al. 2020), as well as mitochondrial DNA (Balloux et al. 2009) and X- and Y-chromosomal loci (Macpherson et al. 2004; Ramachandran et al. 2004, 2008; Shi et al. 2010; Poznik et al. 2013).

While establishing genomic patterns, the panel has also contributed data for understanding the global distribution of variation at particular places in the genome. Examples have focused on specific genes or loci (Thompson et al. 2004; Ferrer-Admetlla et al. 2009; Eisenberg et al. 2010; Ross et al. 2010; Huerta-Sánchez et al. 2014) in an approach facilitated by a general tool for examining SNP allele frequencies in the data (Pickrell et al. 2009). In one example that combines the population-genetic emphasis of the HGDP-CEPH panel with its value for studying individual loci, a microsatellite locus whose distinctive distribution was first noticed in the HGDP-CEPH Native Americans (Zhivotovsky et al. 2003) was later used to understand migration in the Americas (Schroeder et al. 2007, 2009).

**4. Population-genetic statistics, theory, and software.** In applications that extend to population genetics in general, beyond human population genetics, properties of population-genetic statistics have been illustrated in examples with HGDP-CEPH data. Examples have considered mathematical and statistical features of genetic diversity statistics (Rosenberg and Jakobsson 2008; DeGiorgio et al. 2010; Aw and Rosenberg 2018), genetic differentiation statistics (Mountain and Ramakrishnan 2005; Jakobsson et al. 2013; Mehta et al. 2019; Liu et al. 2023), ancestry information content (Rosenberg et al. 2003b; Rosenberg 2005), and linkage disequilibrium (Rosenberg and Blum 2007; Edge et al. 2017). The data have also provided the basis for observations of new population-genetic patterns—for example, that private microsatellite alleles often lie at the extremes of the allele-size distribution—as well as the basis for testing theory that explains those observations (Szpiech and Rosenberg 2011).

In a frequent type of application, population-genetic data analysis methods, both for human data and for the population genetics of diverse species, have used HGDP-CEPH genotypes as test cases in introducing software. In a typical approach, a new algorithm or package is developed—often for measurement, modeling, or inference related to genetic variation, population-genetic structure, genetic ancestry, or spatial genetics at a variety of scales—and its statistical or computational properties are described with the HGDP-CEPH panel supplying the data for an illustration. Examples include ADZE (Szpiech et al. 2008), ALDER (Loh et al. 2013), BAPS2 (Corander et al. 2004), BEDASSLE (Bradburd et al. 2013), DAPC (Jombart et al. 2010), fastSTRUCTURE (Raj et al. 2014), LASER (Wang et al. 2015), lfa (Hao et al. 2016), Locator (Battey et al. 2020), MOSAIC (Salter-Townsend and Myers 2019), mStruct (Shringarpure and Xing 2009), Netstruct (Greenbaum et al. 2019), SelEstim (Vitalis et al. 2014), smartpca (Patterson et al. 2006), SPA (Yang et al. 2012), SpaceMix (Bradburd et al. 2016), SPLATCHE (Ray and Excoffier 2009), and TESS (Francois et al. 2006).

**5. Natural selection, population-level variables, and biomedical studies.** HGDP-CEPH studies have investigated various forms of natural selection, including purifying selection and geographically driven positive selection (Foll and Gaggiotti 2008; Coop et al. 2009; Moreno-Estrada et al. 2009; Xue et al. 2009; Henn et al. 2015). The data have also contributed to comparisons of human genetic variation and variation in pathogens (Linz et al. 2007; Fumagalli et al. 2011), craniometric traits (Roseman 2004; von Cramon-Taubadel 2011), environmental variables (Hancock et al. 2008; Coop et al. 2010; Günther and Coop 2013; Blair et al. 2014), microbiomes (Kidd et al. 2014), and epigenetic and regulatory traits, measured from the HGDP-CEPH cell lines themselves (Martin et al; 2014; Carja et al. 2017; Reynolds and Niedbalski 2024). In one group of studies, the HGDP-CEPH data have been used for testing the concordance of genetic variation with a class of data of great interest to Luca Cavalli-Sforza: linguistic variation (Hunley et al. 2012; Creanza et al. 2015; Verdu et al. 2017).

By examining allele frequencies at phenotype-associated loci, HGDP-CEPH data have contributed insights into geographic variation in genetics of biomedical phenotypes (Myles et al. 2008; Ding and Kullo 2011; Chen et al. 2012; Berg and Coop 2014). The early genomic data from the HGDP-CEPH panel have also been important in evaluating the scientific potential of data collection across many populations, often in biomedical contexts. HGDP-CEPH data have been used in assessing genotype imputation accuracy based on genomic reference panels (Huang et al. 2009a,b; Li et al. 2010), evaluating population representation in embryonic stem-cell research (Mosher et al. 2010), and exploring the potential of diversifying the populations used in association studies (Rosenberg et al. 2010). A notable example was in the area of tag-SNP portability for genome-wide association studies. When the HapMap resource preparing for genome-wide association studies had considered only four populations, González-Néira et al. (2006) and Conrad et al. (2006) evaluated the potential for genomic SNPs chosen based on the four HapMap populations to detect linkage disequilibrium with potential disease loci in other populations, supporting the general utility of the HapMap populations and motivating deeper sampling in Africa.

**6. Visualizations and education.** Studies of the HGDP-CEPH panel have used the data for educational purposes, and new forms of visualization of genetic variation have been developed with data from the panel. These include the bar-plot representation of mixed-membership clustering (Rosenberg et al. 2002), the “common-ancestry profile” representation of genetic similarities of individuals (Mountain and Ramakrishnan 2005), various displays of haplotype patterns (Conrad et al. 2006; Jakobsson et al. 2008; Lawson et al. 2012; San Lucas et al. 2012), rarefaction plots for alleles private to groups of populations (Szpiech et al. 2008), Procrustes rotations of multidimensional scaling and principal components plots of genetic variation (Wang et al. 2010), and new types of tree diagram (Pickrell and Pritchard 2012; Greenbaum et al. 2019).

The HGDP-CEPH data have also been important in summaries that have a broader educational focus than typical scientific articles. These have emphasized the role of Africa in human evolution (McClellan et al. 2017), described the connection of human genetic diversity to biomedical and other practical problems that relate to variation across populations (Cavalli-Sforza 2007; Rosenberg and Kang 2015), and answered frequently asked questions about human genetic diversity (Barbujani and Colonna 2010; Rosenberg 2011).

## References

[iyaf273-B1] Algee-Hewitt BFB, Edge MD, Kim J, Li JZ, Rosenberg NA. 2016. Individual identifiability predicts population identifiability in forensic microsatellite markers. Curr Biol. 26:935–942. 10.1016/j.cub.2016.01.065. 26996508

[iyaf273-B2] Almarri MA et al 2020. Population structure, stratification, and introgression of human structural variation. Cell. 182:189–199.e15. 10.1016/j.cell.2020.05.024. 32531199 PMC7369638

[iyaf273-B3] Ammerman AJ, Cavalli-Sforza LL. 1979. The wave of advance model for the spread of agriculture in Europe. In: Renfrew C, Cooke KL, editors. Transformations: mathematical approaches to culture change. Academic Press. p. 275–293.

[iyaf273-B4] Aneli S, Birolo G, Matullo G. 2022. Twenty years of the Human Genome Diversity Project. Hum Popul Genet Genom. 2:0005. 10.47248/hpgg2202040005.

[iyaf273-B5] Aw AJ, Rosenberg NA. 2018. Bounding measures of genetic similarity and diversity using majorization. J Math Biol. 77:711–737. 10.1007/s00285-018-1226-x. 29569105 PMC6133051

[iyaf273-B6] Balloux F, Lawson Handley LJ, Jombart T, Liu H, Manica A. 2009. Climate shaped the worldwide distribution of human mitochondrial DNA sequence variation. Proc R Soc Lond B Biol Sci. 276:3447–3455. 10.1098/rspb.2009.0752. PMC2817182 19586946

[iyaf273-B7] Barbujani G, Colonna V. 2010. Human genome diversity: frequently asked questions. Trends Genet. 26:285–295. 10.1016/j.tig.2010.04.002. 20471132

[iyaf273-B8] Barbujani G, Magagni A, Minch E, Cavalli-Sforza LL. 1997. An apportionment of human DNA diversity. Proc Natl Acad Sci U S A. 94:4516–4519. 10.1073/pnas.94.9.4516. 9114021 PMC20754

[iyaf273-B9] Bastos-Rodrigues L, Pimenta JR, Pena SDJ. 2006. The genetic structure of human populations studied through short insertion-deletion polymorphisms. Ann Hum Genet. 70:658–665. 10.1111/j.1469-1809.2006.00287.x. 29488221

[iyaf273-B10] Basu A, Sarkar-Roy N, Majumder PP. 2016. Genomic reconstruction of the history of extant populations of India reveals five distinct ancestral components and a complex structure. Proc Natl Acad Sci U S A. 113:1594–1599. 10.1073/pnas.1513197113. 26811443 PMC4760789

[iyaf273-B11] Battey CJ, Ralph PL, Kern AD. 2020. Predicting geographic location from genetic variation with deep neural networks. eLife. 9:e54507. 10.7554/eLife.54507. 32511092 PMC7324158

[iyaf273-B12] Beck CR et al 2010. LINE-1 retrotransposition activity in human genomes. Cell. 141:1159–1170. 10.1016/j.cell.2010.05.021. 20602998 PMC3013285

[iyaf273-B13] Berardinelli GN et al 2022. Association of microsatellite instability (MSI) status with the 5-year outcome and genetic ancestry in a large Brazilian cohort of colorectal cancer. Eur J Hum Genet. 30:824–832. 10.1038/s41431-022-01104-y. 35474354 PMC9259739

[iyaf273-B14] Berg JJ, Coop G. 2014. A population genetic signal of polygenic adaptation. PLoS Genet. 10:e1004412. 10.1371/journal.pgen.1004412. 25102153 PMC4125079

[iyaf273-B15] Bergström A et al 2017. A Neolithic expansion, but strong genetic structure, in the independent history of New Guinea. Science. 357:1160–1163. 10.1126/science.aan3842. 28912245 PMC5802383

[iyaf273-B16] Bergström A et al 2020. Insights into human genetic variation and population history from 929 diverse genomes. Science. 367:eaay5012. 10.1126/science.aay5012. 32193295 PMC7115999

[iyaf273-B17] Blair LM, Feldman MW. 2015. The role of climate and out-of-Africa migration in the frequencies of risk alleles for 21 human diseases. BMC Genet. 16:81. 10.1186/s12863-015-0239-3. 26170196 PMC4501093

[iyaf273-B18] Blair LM, Granka JM, Feldman MW. 2014. On the stability of the Bayenv method in assessing human SNP-environment associations. Hum Genomics. 8:1. 10.1186/1479-7364-8-1. 24405978 PMC3896655

[iyaf273-B19] Bosch E et al 2009. Decay of linkage disequilibrium within genes across HGDP-CEPH human samples: most population isolates do not show increased LD. BMC Genomics. 10:338. 10.1186/1471-2164-10-338. 19638193 PMC2723139

[iyaf273-B20] Botstein D, White RL, Skolnick M, Davis RW. 1980. Construction of a genetic linkage map in man using restriction fragment length polymorphisms. Am J Hum Genet. 32:314–331. 6247908 PMC1686077

[iyaf273-B21] Bowcock AM et al 1991. Drift, admixture, and selection in human evolution: a study with DNA polymorphisms. Proc Natl Acad Sci U S A. 88:839–843. 10.1073/pnas.88.3.839. 1992475 PMC50909

[iyaf273-B22] Bowcock AM et al 1994. High resolution of human evolutionary trees with polymorphic microsatellites. Nature. 368:455–457. 10.1038/368455a0. 7510853

[iyaf273-B23] Bradburd GS, Ralph PL, Coop GM. 2013. Disentangling the effects of geographic and ecological isolation on genetic differentiation. Evolution. 67:3258–3273. 10.1111/evo.12193. 24102455 PMC3808528

[iyaf273-B24] Bradburd GS, Ralph PL, Coop GM. 2016. A spatial framework for understanding population structure and admixture. PLoS Genet. 12:e1005703. 10.1371/journal.pgen.1005703. 26771578 PMC4714911

[iyaf273-B25] Bryc K et al 2010. Genome-wide patterns of population structure and admixture among Hispanic/Latino populations. Proc Natl Acad Sci U S A. 107:8954–8961. 10.1073/pnas.0914618107. 20445096 PMC3024022

[iyaf273-B26] Cann HM . 1998. Human genome diversity. C R Acad Sci III. 321:443–446. 10.1016/S0764-4469(98)80774-9. 9769857

[iyaf273-B27] Cann HM et al 2002. A human genome diversity cell line panel. Science. 296:261–262. 10.1126/science.296.5566.261b. 11954565

[iyaf273-B28] Cann RL, Stoneking M, Wilson AC. 1987. Mitochondrial DNA and human evolution. Nature. 325:31–36. 10.1038/325031a0. 3025745

[iyaf273-B29] Carja O et al 2017. Worldwide patterns of human epigenetic variation. Nat Ecol Evol. 1:1577–1583. 10.1038/s41559-017-0299-z. 29185505 PMC7580032

[iyaf273-B30] Cavalli-Sforza LL . 1997. Human genome diversity: where is the project now? In: Knoppers BM, Laberge CM, Hirtle M, editors. Human DNA: law and policy. Kluwer Law International. p. 219–227.

[iyaf273-B31] Cavalli-Sforza LL . 2005. The Human Genome Diversity Project: past, present and future. Nat Rev Genet. 6:333–340. 10.1038/nrg1579. 15803201

[iyaf273-B32] Cavalli-Sforza LL . 2007. Human evolution and its relevance for genetic epidemiology. Annu Rev Genomics Hum Genet. 8:1–15. 10.1146/annurev.genom.8.080706.092403. 17408354

[iyaf273-B33] Cavalli-Sforza LL, Bodmer WF, Dausset J. 1997. Support for genetic diversity project. Nature. 390:221. 10.1038/36711. 9384364

[iyaf273-B34] Cavalli-Sforza LL, Menozzi P, Piazza A. 1994. The history and geography of human genes. Princeton University Press.

[iyaf273-B35] Cavalli-Sforza LL, Moroni A, Zei G. 2004. Consanguinity, inbreeding, and genetic drift in Italy. Princeton University Press.

[iyaf273-B36] Cavalli-Sforza LL, Wilson AC, Cantor CR, Cook-Deegan RM, King M-C. 1991. Call for a worldwide survey of human genetic diversity: a vanishing opportunity for the Human Genome Project. Genomics. 11:490–491. 10.1016/0888-7543(91)90169-F. 1769670

[iyaf273-B37] Chen R et al 2012. Type 2 diabetes risk alleles demonstrate extreme directional differentiation among human populations, compared to other diseases. PLoS Genet. 8:e1002621. 10.1371/journal.pgen.1002621. 22511877 PMC3325177

[iyaf273-B38] Collins FS, Guyer MS, Chakravarti A. 1997. Variations on a theme: cataloging human DNA sequence variation. Science. 278:1580–1581. 10.1126/science.278.5343.1580. 9411782

[iyaf273-B39] Committee on Human Genome Diversity . 1997. Evaluating human genome diversity. National Academy of Sciences.

[iyaf273-B40] Conrad DF et al 2006. A worldwide survey of haplotype variation and linkage disequilibrium in the human genome. Nat Genet. 38:1251–1260. 10.1038/ng1911. 17057719

[iyaf273-B41] Coop G et al 2009. The role of geography in human adaptation. PLoS Genet. 5:e1000500. 10.1371/journal.pgen.1000500. 19503611 PMC2685456

[iyaf273-B42] Coop G, Witonsky D, Di Rienzo A, Pritchard JK. 2010. Using environmental correlations to identify loci underlying local adaptation. Genetics. 185:1411–1423. 10.1534/genetics.110.114819. 20516501 PMC2927766

[iyaf273-B43] Corander J, Waldmann P, Marttinen P, Sillanpää MJ. 2004. BAPS 2: enhanced possibilities for the analysis of genetic population structure. Bioinformatics. 20:2363–2369. 10.1093/bioinformatics/bth250. 15073024

[iyaf273-B44] Cousins T, Scally A, Durbin R. 2025. A structured coalescent model reveals deep ancestral structure shared by all modern humans. Nature. 57:856–864. 10.1038/s41588-025-02117-1. PMC11985351 40102687

[iyaf273-B45] Creanza N et al 2015. A comparison of worldwide phonemic and genetic variation in human populations. Proc Natl Acad Sci U S A. 111:3497–3502. 10.1073/pnas.1424033112. PMC4321277 25605893

[iyaf273-B46] DeGiorgio M, Degnan JH, Rosenberg NA. 2011. Coalescence-time distributions in a serial founder model of human evolutionary history. Genetics. 189:579–593. 10.1534/genetics.111.129296. 21775469 PMC3189793

[iyaf273-B47] DeGiorgio M, Jakobsson M, Rosenberg NA. 2009. Explaining worldwide patterns of human genetic variation using a coalescent-based serial founder model of migration outward from Africa. Proc Natl Acad Sci U S A. 106:16057–16062. 10.1073/pnas.0903341106. 19706453 PMC2752555

[iyaf273-B48] DeGiorgio M, Jankovic I, Rosenberg NA. 2010. Unbiased estimation of gene diversity in samples containing related individuals: exact variance and arbitrary ploidy. Genetics. 186:1367–1387. 10.1534/genetics.110.121756. 20923981 PMC2998318

[iyaf273-B49] DeGiorgio M, Rosenberg NA. 2009. An unbiased estimator of gene diversity in samples containing related individuals. Mol Biol Evol. 26:501–512. 10.1093/molbev/msn254. 18988687 PMC2915906

[iyaf273-B50] Ding K, Kullo IJ. 2011. Geographic differences in allele frequencies of susceptibility SNPs for cardiovascular disease. BMC Med Genet. 12:55. 10.1186/1471-2350-12-55. 21507254 PMC3103418

[iyaf273-B51] Edge MD, Algee-Hewitt BFB, Pemberton TJ, Li JZ, Rosenberg NA. 2017. Linkage disequilibrium matches forensic genetic records to disjoint genomic marker sets. Proc Natl Acad Sci U S A. 114:5671–5676. 10.1073/pnas.1619944114. 28507140 PMC5465933

[iyaf273-B52] Eisenberg DTA, Kuzawa CW, Hayes MG. 2010. Worldwide allele frequencies of the human apolipoprotein E gene: climate, local adaptations, and evolutionary history. Am J Phys Anthropol. 143:100–111. 10.1002/ajpa.21298. 20734437

[iyaf273-B53] Excoffier L, Hamilton G. 2003. Comment on “Genetic structure of human populations”. Science. 300:1877. 10.1126/science.1083411. 12817127

[iyaf273-B202] Ferrer-Admetlla A et al 2009. A natural history of FUT2 polymorphism in humans. Mol Biol Evol. 26:1993–2003. 10.1093/molbev/msp108. 19487333

[iyaf273-B54] Fisher RA . 1937. The wave of advance of advantageous genes. Ann Eugen. 7:355–369. 10.1111/j.1469-1809.1937.tb02153.x.

[iyaf273-B55] Foll M, Gaggiotti O. 2008. A genome-scan method to identify selected loci appropriate for both dominant and codominant markers: a Bayesian perspective. Genetics. 180:977–993. 10.1534/genetics.108.092221. 18780740 PMC2567396

[iyaf273-B56] Francois O, Ancelet S, Guillot G. 2006. Bayesian clustering using hidden Markov random fields in spatial population genetics. Genetics. 174:805–816. 10.1534/genetics.106.059923. 16888334 PMC1602073

[iyaf273-B57] Friedlaender JS et al 2008. The genetic structure of Pacific islanders. PLoS Genet. 4:e19. 10.1371/journal.pgen.0040019. 18208337 PMC2211537

[iyaf273-B58] Fumagalli M et al 2011. Signatures of environmental genetic adaptation pinpoint pathogens as the main selective pressure through human evolution. PLoS Genet. 7:e1002355. 10.1371/journal.pgen.1002355. 22072984 PMC3207877

[iyaf273-B59] González-Néira A et al 2006. The portability of tagSNPs across populations: a worldwide survey. Genome Res. 16:323–330. 10.1101/gr.4138406. 16467560 PMC1415211

[iyaf273-B60] Greely HT . 1997. The ethics of the Human Genome Diversity Project: the North American Regional Committee's proposed model ethical protocol. In: Knoppers BM, Laberge CM, Hirtle M, editors. Human DNA: law and policy. Kluwer Law International. p. 239–256.

[iyaf273-B61] Greely HT . 2001. Human genome diversity: what about the other human genome project? Nat Rev Genet. 2:222–227. 10.1038/35056071. 11256074

[iyaf273-B62] Green RE et al 2010. A draft sequence of the Neandertal genome. Science. 328:710–722. 10.1126/science.1188021. 20448178 PMC5100745

[iyaf273-B63] Greenbaum G, Rubin A, Templeton AR, Rosenberg NA. 2019. Network-based hierarchical population structure analysis for large genomic data sets. Genome Res. 29:2020–2033. 10.1101/gr.250092.119. 31694865 PMC6886512

[iyaf273-B64] Grimes BF . 1992, editor. Ethnologue: languages of the world, Vol. 12. Summer Institute of Linguistics.

[iyaf273-B65] Grundler MC, Terhorst J, Bradburd GS. 2025. A geographic history of human genetic ancestry. Science. 387:1391–1397. 10.1126/science.adp4642. 40146820 PMC12132082

[iyaf273-B66] Günther T, Coop G. 2013. Robust identification of local adaptation from allele frequencies. Genetics. 195:205–220. 10.1534/genetics.113.152462. 23821598 PMC3761302

[iyaf273-B67] Hall JM et al 1990. Linkage of early-onset familial breast cancer to chromosome 17q21. Science. 250:1684–1689. 10.1126/science.2270482. 2270482

[iyaf273-B68] Hancock AM et al 2008. Adaptations to climate in candidate genes for common metabolic disorders. PLoS Genet. 4:e32. 10.1371/journal.pgen.0040032. 18282109 PMC2242814

[iyaf273-B69] Hao W, Song M, Storey JD. 2016. Probabilistic models of genetic variation in structured populations applied to global human studies. Bioinformatics. 32:713–721. 10.1093/bioinformatics/btv641. 26545820 PMC4795615

[iyaf273-B70] Hellenthal G et al 2014. A genetic atlas of human admixture history. Science. 343:747–751. 10.1126/science.1243518. 24531965 PMC4209567

[iyaf273-B71] Henn BM et al 2011. Hunter-gatherer genomic diversity suggests a southern African origin for modern humans. Proc Natl Acad Sci U S A. 108:5154–5162. 10.1073/pnas.1017511108. 21383195 PMC3069156

[iyaf273-B72] Henn BM et al 2012. Cryptic distant relatives are common in both isolated and cosmopolitan genetic samples. PLoS One. 7:e34267. 10.1371/journal.pone.0034267. 22509285 PMC3317976

[iyaf273-B73] Henn BM, Boutigué LR, Peischl S, Bustamante CD. 2015. Distance from sub-Saharan Africa predicts mutational load in diverse human genomes. Proc Natl Acad Sci U S A. 113:E440–E449. 10.1073/pnas.1510805112. 26712023 PMC4743782

[iyaf273-B74] Hofer T, Ray N, Wegmann D, Excoffier L. 2009. Large allele frequency differences between human continental groups are more likely to have occurred by drift during range expansions than by selection. Ann Hum Genet. 73:95–108. 10.1111/j.1469-1809.2008.00489.x. 19040659

[iyaf273-B75] Hollenbach JA, Nocedal I, Ladner MB, Single RM, Trachtenberg EA. 2012. Killer cell immunoglobulin-like receptor (KIR) gene content variation in the HGDP-CEPH populations. Immunogenetics. 64:719–737. 10.1007/s00251-012-0629-x. 22752190 PMC3438391

[iyaf273-B76] Hu W et al 2023. Genomic inference of a severe human bottleneck during the Early to Middle Pleistocene transition. Science. 381:979–984. 10.1126/science.abq7487. 37651513

[iyaf273-B77] Huang L et al 2009a. Genotype-imputation accuracy across worldwide human populations. Am J Hum Genet. 84:235–250. 10.1016/j.ajhg.2009.01.013. 19215730 PMC2668016

[iyaf273-B78] Huang L et al 2009b. The relationship between imputation error and statistical power in genetic association studies in diverse populations. Am J Hum Genet. 85:692–698. 10.1016/j.ajhg.2009.09.017. 19853241 PMC2775841

[iyaf273-B79] Huerta-Sánchez E et al 2014. Altitude adaptation in Tibetans caused by introgression of Denisovan-like DNA. Nature. 512:194–197. 10.1038/nature13408. 25043035 PMC4134395

[iyaf273-B80] Hunley KL, Cabana GS, Long JC. 2016. The apportionment of human diversity revisited. Am J Phys Anthropol. 160:561–569. 10.1002/ajpa.22899. 26619959

[iyaf273-B81] Hunley KL, Healy M, Long JC. 2012. The impact of founder effects, gene flow, and European admixture on native American genetic diversity. Proc Biol Sci. 279:295–302. 10.1002/ajpa.21506. 21913174

[iyaf273-B82] Itsara A et al 2009. Population analysis of large copy number variants and hotspots of human genetic disease. Am J Hum Genet. 84:148–161. 10.1016/j.ajhg.2008.12.014. 19166990 PMC2668011

[iyaf273-B83] Jakobsson M et al 2008. Genotype, haplotype and copy-number variation in worldwide human populations. Nature. 451:998–1003. 10.1038/nature06742. 18288195

[iyaf273-B84] Jakobsson M, Edge MD, Rosenberg NA. 2013. The relationship between *F*_ST_ and the frequency of the most frequent allele. Genetics. 193:515–528. 10.1534/genetics.112.144758. 23172852 PMC3567740

[iyaf273-B85] Jay F, Sjödin P, Jakobsson M, Blum MGB. 2013. Anisotropic isolation by distance: the main orientations of human genetic differentiation. Mol Biol Evol. 30:513–525. 10.1093/molbev/mss259. 23171862 PMC3563970

[iyaf273-B86] Jombart T, Devillard S, Balloux F. 2010. Discriminant analysis of principal components: a new method for the analysis of genetically structured populations. BMC Genet. 11:94. 10.1186/1471-2156-11-94. 20950446 PMC2973851

[iyaf273-B87] Kerdoncuff E et al 2025. 50,000 years of evolutionary history of India: impact on health and disease variation. Cell. 188:3389–3404.e6. 10.1016/j.cell.2025.04.027. 40578318 PMC12376946

[iyaf273-B88] Kevles DJ, Hood L. 1992. The code of codes: scientific and social issues in the Human Genome Project. Harvard University Press. 10.1038/358027a0 11643003

[iyaf273-B89] Kidd JM et al 2014. Exome capture from saliva produces high quality genomic and metagenomic data. BMC Genomics. 15:262. 10.1186/1471-2164-15-262. 24708091 PMC4051168

[iyaf273-B90] Kimura M, Weiss GH. 1964. The stepping stone model of population structure and the decrease of genetic correlation with distance. Genetics. 49:561–576. 10.1093/genetics/49.4.561. 17248204 PMC1210594

[iyaf273-B91] Kirin M et al 2010. Genomic runs of homozygosity record population history and consanguinity. PLoS One. 5:e13996. 10.1371/journal.pone.0013996. 21085596 PMC2981575

[iyaf273-B92] Knoppers BM, Laberge CM, Hirtle M. 1997, editors. Human DNA: law and policy. Kluwer Law International.

[iyaf273-B93] Koenig Z et al 2024. A harmonized public resource of deeply sequenced diverse human genomes. Genome Res. 34:796–809. 10.1101/gr.278378.123. 38749656 PMC11216312

[iyaf273-B94] Lawson DJ, Hellenthal G, Myers S, Falush D. 2012. Inference of population structure using dense haplotype data. PLoS Genet. 8:e1002453. 10.1371/journal.pgen.1002453. 22291602 PMC3266881

[iyaf273-B95] Lawson Handley LJ, Manica A, Goudet J, Balloux F. 2007. Going the distance: human population genetics in a clinal world. Trends Genet. 23:432–439. 10.1016/j.tig.2007.07.002. 17655965

[iyaf273-B96] Leutenegger AL, Sahbatou M, Gazal S, Cann H, Génin E. 2011. Consanguinity around the world: what do the genomic data of the HGDP-CEPH diversity panel tell US? Eur J Hum Genet. 19:583–587. 10.1038/ejhg.2010.205. 21364699 PMC3083608

[iyaf273-B97] Lewontin RC . 1972. The apportionment of human diversity. Evol Biol. 6:381–398.

[iyaf273-B98] Li JZ et al 2008. Worldwide human relationships inferred from genome-wide patterns of variation. Science. 319:1100–1104. 10.1126/science.1153717. 18292342

[iyaf273-B99] Li Y, Willer CJ, Ding J, Scheet P, Abecasis GR. 2010. MaCH: using sequence and genotype data to estimate haplotypes and unobserved genotypes. Genet Epidemiol. 34:816–834. 10.1002/gepi.20533. 21058334 PMC3175618

[iyaf273-B100] Lieberman MV, Montgomery DW. 1988. First-mover advantages. Strateg Manag J. 9:41–58. 10.1002/smj.4250090706.

[iyaf273-B101] Linz BL et al 2007. An African origin for the intimate association between humans and *Helicobacter pylori*. Nature. 445:915–918. 10.1038/nature05562. 17287725 PMC1847463

[iyaf273-B102] Liu X, Ahsan Z, Martheswaran TK, Rosenberg NA. 2023. When is the allele-sharing dissimilarity between two populations exceeded by the allele-sharing dissimilarity of a population with itself? Stat Appl Genet Mol Biol. 22:2023004. 10.1515/sagmb-2023-0004. PMC10711674 38073574

[iyaf273-B103] Liu H, Prugnolle F, Manica A, Balloux F. 2006. A geographically explicit genetic model of worldwide human-settlement history. Am J Hum Genet. 79:230–237. 10.1086/505436. 16826514 PMC1559480

[iyaf273-B104] Loh PR et al 2013. Inferring admixture histories of human populations using linkage disequilibrium. Genetics. 193:1233–1254. 10.1534/genetics.112.147330. 23410830 PMC3606100

[iyaf273-B105] López Herráez D et al 2009. Genetic variation and recent positive selection in worldwide human populations: evidence from nearly 1 million SNPs. PLoS One. 4:e7888. 10.1371/journal.pone.0007888. 19924308 PMC2775638

[iyaf273-B106] Macpherson JM, Ramachandran S, Diamond L, Feldman MW. 2004. Demographic estimates from Y chromosome microsatellite polymorphisms: analysis of a worldwide sample. Hum Genomics. 1:345–354. 10.1186/1479-7364-1-5-345. 15588495 PMC3525096

[iyaf273-B107] Mallick S et al 2016. The Simons Genome Diversity Project: 300 genomes from 142 diverse populations. Nature. 538:201–206. 10.1038/nature18964. 27654912 PMC5161557

[iyaf273-B108] Manichaikul A et al 2012. Population structure of Hispanics in the United States: the multi-ethnic study of atherosclerosis. PLoS Genet. 8:e1002640. 10.1371/journal.pgen.1002640. 22511882 PMC3325201

[iyaf273-B109] Martin AR et al 2014. Transcriptome sequencing from diverse human populations reveals differentiated regulatory architecture. PLoS Genet. 10:e1004549. 10.1371/journal.pgen.1004549. 25121757 PMC4133153

[iyaf273-B110] McClellan JM, Lehner T, King MC. 2017. Gene discovery for complex traits: lessons from Africa. Cell. 171:261–264. 10.1016/j.cell.2017.09.037. 28985555

[iyaf273-B111] M’charek A . 2005. The Human Genome Diversity Project: an ethnography of scientific practice. Cambridge University Press.

[iyaf273-B112] McIlwain C . 1997. Diversity project ‘does not merit federal funding’. Nature. 389:774. 10.1038/39693. 9349796

[iyaf273-B113] Mehta RS, Feder AF, Boca SM, Rosenberg NA. 2019. The relationship between haplotype-based *F*_ST_ and haplotype length. Genetics. 213:281–295. 10.1534/genetics.119.302430. 31285255 PMC6727796

[iyaf273-B114] Moorjani P et al 2013. Genetic evidence for recent population mixture in India. Am J Hum Genet. 93:422–438. 10.1016/j.ajhg.2013.07.006. 23932107 PMC3769933

[iyaf273-B115] Moreno-Estrada A et al 2009. Interrogating 11 fast-evolving genes for signatures of recent positive selection in worldwide human populations. Mol Biol Evol. 26:2285–2297. 10.1093/molbev/msp134. 19578157

[iyaf273-B116] Moreno-Estrada A et al 2013. Reconstructing the population genetic history of the Caribbean. PLoS Genet. 9:e1003925. 10.1371/journal.pgen.1003925. 24244192 PMC3828151

[iyaf273-B117] Mosher JT et al 2010. Lack of population diversity in commonly used human embryonic stem-cell lines. N Engl J Med. 362:183–185. 10.1056/NEJMc0910371. 20018958

[iyaf273-B118] Mountain JL, Ramakrishnan U. 2005. Impact of human population history on distributions of individual-level genetic distance. Hum Genomics. 2:4–19. 10.1186/1479-7364-2-1-4. 15814064 PMC3525116

[iyaf273-B119] Myles S, Davison D, Barrett J, Stoneking M, Timpson N. 2008. Worldwide population differentiation at disease-associated SNPs. BMC Med Genomics. 1:22. 10.1186/1755-8794-1-22. 18533027 PMC2440747

[iyaf273-B120] North American Regional Committee of the Human Genome Diversity Project . 1997. Proposed model ethical protocol for collecting DNA samples. Houst Law Rev. 33:1431–1473. 12627556

[iyaf273-B121] Patterson N et al 2012. Ancient admixture in human history. Genetics. 192:1065–1093. 10.1534/genetics.112.145037. 22960212 PMC3522152

[iyaf273-B122] Patterson N, Price AL, Reich D. 2006. Population structure and eigenanalysis. PLoS Genet. 2:e190. 10.1371/journal.pgen.0020190. 17194218 PMC1713260

[iyaf273-B123] Pemberton TJ et al 2012. Genomic patterns of homozygosity in worldwide human populations. Am J Hum Genet. 91:275–292. 10.1016/j.ajhg.2012.06.014. 22883143 PMC3415543

[iyaf273-B124] Pemberton TJ, DeGiorgio M, Rosenberg NA. 2013. Population structure in a comprehensive genomic data set on human microsatellite variation. G3 (Bethesda). 3:891–907. 10.1534/g3.113.005728. 23550135 PMC3656735

[iyaf273-B125] Pemberton TJ, Rosenberg NA. 2014. Population-genetic influences on genomic estimates of the inbreeding coefficient: a global perspective. Hum Hered. 77:37–48. 10.1159/000362878. 25060268 PMC4154368

[iyaf273-B126] Pemberton TJ, Sandefur CI, Jakobsson M, Rosenberg NA. 2009. Sequence determinants of human microsatellite variability. BMC Genomics. 10:612. 10.1186/1471-2164-10-612. 20015383 PMC2806349

[iyaf273-B203] Pennisi E . 1997. NRC OKs long-delayed survey of human henome diversity. Science. 278:568. 10.1126/science.278.5338.568. 9381161

[iyaf273-B127] Pickrell JK et al 2009. Signals of recent positive selection in a worldwide sample of human populations. Genome Res. 19:826–837. 10.1101/gr.087577.108. 19307593 PMC2675971

[iyaf273-B128] Pickrell JK, Pritchard JK. 2012. Inference of population splits and mixtures from genome-wide allele frequency data. PLoS Genet. 8:e1002967. 10.1371/journal.pgen.1002967. 23166502 PMC3499260

[iyaf273-B129] Poznik GD et al 2013. Sequencing Y chromosomes resolves discrepancy in time to common ancestor of males versus females. Science. 341:562–565. 10.1126/science.1237619. 23908239 PMC4032117

[iyaf273-B130] Prates L, Lemes RB, Hünemeier T, Leonardi F. 2023. Population-based change-point detection for the identification of homozygosity islands. Bioinformatics. 39:btad170. 10.1093/bioinformatics/btad170. 37039826 PMC10112956

[iyaf273-B131] Prugnolle F, Manica A, Balloux F. 2005. Geography predicts neutral genetic diversity of human populations. Curr Biol. 15:R159–R160. 10.1016/j.cub.2005.02.038. 15753023 PMC1800886

[iyaf273-B132] Raghavan M et al 2015. Genomic evidence for the Pleistocene and recent population history of native Americans. Science. 349:aab3884. 10.1126/science.aab3884. 26198033 PMC4733658

[iyaf273-B133] Raj A, Stephens M, Pritchard JK. 2014. fastSTRUCTURE: variational inference of population structure in large SNP data sets. Genetics. 197:573–589. 10.1534/genetics.114.164350. 24700103 PMC4063916

[iyaf273-B134] Ramachandran S et al 2005. Support from the relationship of genetic and geographic distance in human populations for a serial founder effect originating in Africa. Proc Natl Acad Sci U S A. 102:15942–15947. 10.1073/pnas.0507611102. 16243969 PMC1276087

[iyaf273-B135] Ramachandran S, Rosenberg NA. 2011. A test of the influence of continental axes of orientation on patterns of human gene flow. Am J Phys Anthropol. 146:515–529. 10.1002/ajpa.21533. 21913175 PMC3538872

[iyaf273-B136] Ramachandran S, Rosenberg NA, Feldman MW, Wakeley J. 2008. Population differentiation and migration: coalescence times in a two-sex island model for autosomal and X-linked loci. Theor Popul Biol. 74:291–301. 10.1016/j.tpb.2008.08.003. 18817799 PMC2630000

[iyaf273-B137] Ramachandran S, Rosenberg NA, Zhivotovsky LA, Feldman MW. 2004. Robustness of the inference of human population structure: a comparison of X-chromosomal and autosomal microsatellites. Hum Genomics. 1:87–97. 10.1186/1479-7364-1-2-87. 15601537 PMC3525066

[iyaf273-B138] Rasmussen M et al 2011. An aboriginal Australian genome reveals separate human dispersals into Asia. Science. 334:94–98. 10.1126/science.1211177. 21940856 PMC3991479

[iyaf273-B139] Ray N, Excoffier L. 2009. Inferring past demography using spatially explicit population genetic models. Hum Biol. 81:141–157. 10.1353/hub.2009.a362932. 19943741

[iyaf273-B140] Reardon J . 2001. The Human Genome Diversity Project: a case study in coproduction. Soc Stud Sci. 31:357–388. 10.1177/030631201031003002.

[iyaf273-B141] Reardon J . 2005. Race to the finish: identity and governance in an age of genomics. Princeton University Press.

[iyaf273-B142] Reich D et al 2010. Genetic history of an archaic hominin group from Denisova Cave in Siberia. Nature. 468:1053–1060. 10.1038/nature09710. 21179161 PMC4306417

[iyaf273-B143] Reich D et al 2012. Reconstructing native American population history. Nature. 488:370–374. 10.1038/nature11258. 22801491 PMC3615710

[iyaf273-B144] Resnik DB . 1999. The Human Genome Diversity Project: ethical problems and solutions. Politics Life Sci. 18:15–23. 10.1017/S0730938400023510. 11660815

[iyaf273-B145] Reynolds AZ, Niedbalski SD. 2024. Sex-biased gene regulation varies across human populations as a result of adaptive evolution. Am J Biol Anthropol. 183:e24888. 10.1002/ajpa.24888. 38100225 PMC11279473

[iyaf273-B146] Roberts L . 1991. A genetic survey of vanishing peoples. Science. 252:1614–1617. 10.1126/science.2047869. 2047869

[iyaf273-B147] Roseman CC . 2004. Detecting interregionally diversifying natural selection on modern human cranial form by using matched molecular and morphometric data. Proc Natl Acad Sci U S A. 101:12824–12829. 10.1073/pnas.0402637101. 15326305 PMC516480

[iyaf273-B148] Rosenberg NA et al 2002. Genetic structure of human populations. Science. 298:2381–2385. 10.1126/science.1078311. 12493913

[iyaf273-B149] Rosenberg NA et al 2003a. Response to comment on “Genetic structure of human populations”. Science. 300:1877. 10.1126/science.1084688. 12817127

[iyaf273-B150] Rosenberg NA . 2005. Algorithms for selecting informative marker panels for population assignment. J Comput Biol. 12:1183–1201. 10.1089/cmb.2005.12.1183. 16305328

[iyaf273-B151] Rosenberg NA et al 2005. Clines, clusters, and the effect of study design on the inference of human population structure. PLoS Genet. 1:e70. 10.1371/journal.pgen.0010070. 16355252 PMC1310579

[iyaf273-B152] Rosenberg NA et al 2006. Low levels of genetic divergence across geographically and linguistically diverse populations from India. PLoS Genet. 2:e215. 10.1371/journal.pgen.0020215. 17194221 PMC1713257

[iyaf273-B153] Rosenberg NA . 2006. Standardized subsets of the HGDP-CEPH Human Genome Diversity Cell Line Panel, accounting for atypical and duplicated samples and pairs of close relatives. Ann Hum Genet. 70:841–847. 10.1111/j.1469-1809.2006.00285.x. 17044859

[iyaf273-B154] Rosenberg NA et al 2010. Genome-wide association studies in diverse populations. Nat Rev Genet. 11:356–366. 10.1038/nrg2760. 20395969 PMC3079573

[iyaf273-B155] Rosenberg NA . 2011. A population-genetic perspective on the similarities and differences among worldwide human populations. Hum Biol. 83:659–684. 10.1353/hub.2011.a465110. 22276967 PMC3531797

[iyaf273-B156] Rosenberg NA, Blum MGB. 2007. Sampling properties of homozygosity-based statistics for linkage disequilibrium. Math Biosci. 208:33–47. 10.1016/j.mbs.2006.07.001. 17157882

[iyaf273-B157] Rosenberg NA, Jakobsson M. 2008. The relationship between homozygosity and the frequency of the most frequent allele. Genetics. 179:2027–2036. 10.1534/genetics.107.084772. 18689892 PMC2516077

[iyaf273-B204] Rosenberg NA, Kang JTL. 2015. Genetic diversity and societally important disparities. Genetics. 201:1–12. 10.1534/genetics.115.176750. 26354973 PMC4566256

[iyaf273-B158] Rosenberg NA, Li LM, Ward R, Pritchard JK. 2003b. Informativeness of genetic markers for inference of ancestry. Am J Hum Genet. 73:1402–1422. 10.1086/380416. 14631557 PMC1180403

[iyaf273-B159] Ross KA et al 2010. Worldwide allele frequency distribution of four polymorphisms associated with warfarin dose requirements. J Hum Genet. 55:582–589. 10.1038/jhg.2010.73. 20555338

[iyaf273-B160] Salopek P . 1997a. Basically, we are all the same. *Chicago Tribune*. April 27, 1997.

[iyaf273-B161] Salopek P . 1997b. Genes offer sampling of hope and fear. *Chicago Tribune.* April 28, 1997.

[iyaf273-B162] Salter-Townsend M, Myers S. 2019. Fine-scale inference of ancestry segments without prior knowledge of admixing groups. Genetics. 212:869–889. 10.1534/genetics.119.302139. 31123038 PMC6614886

[iyaf273-B163] San Lucas FA, Rosenberg NA, Scheet P. 2012. Haploscope: a tool for the graphical display of haplotype structure in populations. Genet Epidemiol. 36:17–21. 10.1002/gepi.20640. 22147662 PMC3725725

[iyaf273-B164] Santos C . 2015. Completion of a worldwide reference panel of samples for an ancestry informative indel assay. Forensic Sci Int Genet. 17:75–80. 10.1016/j.fsigen.2015.03.011. 25840342

[iyaf273-B165] Schlebusch CM et al 2012. Genomic variation in seven Khoe-San groups reveals adaptation and complex African history. Science. 338:374–379. 10.1126/science.1227721. 22997136 PMC8978294

[iyaf273-B166] Schroeder KB et al 2007. A private allele ubiquitous in the Americas. Biol Lett. 3:218–223. 10.1098/rsbl.2006.0609. 17301009 PMC2375964

[iyaf273-B167] Schroeder KB et al 2009. The distribution of human mitochondrial DNA diversity in the Americas. Mol Biol Evol. 26:1–14.18984901

[iyaf273-B168] Schull WJ . 1997. Support for genetic diversity project. Nature. 390:221. 10.1038/36711. 9384363

[iyaf273-B169] Schull WJ . 1998. Genetic diversity survey. Science. 279:10–15. 10.1126/science.279.5347.10h. 9441399

[iyaf273-B170] Serre D, Pääbo S. 2004. Evidence for gradients of human genetic diversity within and among continents. Genome Res. 14:1679–1685. 10.1101/gr.2529604. 15342553 PMC515312

[iyaf273-B171] Shi W et al 2010. A worldwide survey of human male demographic history based on Y-SNP and Y-STR data from the HGDP-CEPH populations. Mol Biol Evol. 27:385–393. 10.1093/molbev/msp243. 19822636 PMC2806244

[iyaf273-B172] Shringarpure S, Xing EP. 2009. mStruct: inference of population structure in light of both genetic admixing and allele mutations. Genetics. 182:575–593. 10.1534/genetics.108.100222. 19363128 PMC2691765

[iyaf273-B173] Stone L, Lurquin PF. 2005. A genetic and cultural odyssey: the life and work of L. Luca Cavalli-Sforza. Columbia University Press.

[iyaf273-B174] Sümer AP et al 2025. Earliest modern human genomes constrain timing of Neanderthal admixture. Nature. 638:711–717. 10.1038/s41586-024-08420-x. 39667410 PMC11839475

[iyaf273-B175] Szpiech ZA et al 2013. Long runs of homozygosity are enriched for deleterious variation. Am J Hum Genet. 93:90–102. 10.1016/j.ajhg.2013.05.003. 23746547 PMC3710769

[iyaf273-B176] Szpiech ZA, Jakobsson M, Rosenberg NA. 2008. ADZE: a rarefaction approach for counting alleles private to combinations of populations. Bioinformatics. 24:2498–2504. 10.1093/bioinformatics/btn478. 18779233 PMC2732282

[iyaf273-B177] Szpiech ZA, Rosenberg NA. 2011. On the size distribution of private microsatellite alleles. Theor Popul Biol. 80:100–113. 10.1016/j.tpb.2011.03.006. 21514313 PMC3143247

[iyaf273-B178] Taubes G . 1995. Scientists attacked for ‘patenting’ Pacific tribe. Science. 270:1112. 10.1126/science.270.5239.1112. 7502030

[iyaf273-B179] Thompson EE et al 2004. CYP3A variation and the evolution of salt-sensitivity variants. Am J Hum Genet. 75:1059–1069. 10.1086/426406. 15492926 PMC1182141

[iyaf273-B180] Thornton TA, Bermejo JL. 2014. Local and global ancestry inference and applications to genetic association analysis for admixed populations. Genet Epidemiol. 38:S5–S12. 10.1002/gepi.21819. 25112189 PMC4339867

[iyaf273-B181] Tishkoff SA et al 2009. The genetic structure and history of Africans and African Americans. Science. 324:1035–1044. 10.1126/science.1172257. 19407144 PMC2947357

[iyaf273-B182] Verdu P, Jewett EM, Pemberton TJ, Rosenberg NA, Baptista M. 2017. Parallel trajectories of genetic and linguistic admixture in a genetically admixed Creole population. Curr Biol. 27:2529–2535.e3. 10.1016/j.cub.2017.07.002. 28803872

[iyaf273-B183] Vitalis R, Gautier M, Dawson KJ, Beaumont MA. 2014. Detecting and measuring selection from gene frequency data. Genetics. 196:799–817. 10.1534/genetics.113.152991. 24361938 PMC3948807

[iyaf273-B184] von Cramon-Taubadel N . 2011. The relative efficacy of functional and developmental cranial modules for reconstructing global human population history. Am J Phys Anthropol. 146:83–93. 10.1002/ajpa.21550. 21710659

[iyaf273-B185] Wang S et al 2007. Genetic variation and population structure in Native Americans. PLoS Genet. 3:e185. 10.1371/journal.pgen.0030185. 18039031 PMC2082466

[iyaf273-B186] Wang S et al 2008. Geographic patterns of genome admixture in Latin American Mestizos. PLoS Genet. 4:e1000037. 10.1371/journal.pgen.1000037. 18369456 PMC2265669

[iyaf273-B187] Wang C et al 2010. Comparing spatial maps of human population-genetic variation using Procrustes analysis. Stat Appl Genet Mol Biol. 9:13. 10.2202/1544-6115.1493. 20196748 PMC2861313

[iyaf273-B188] Wang C, Zhan X, Liang L, Abecasis GR, Lin X. 2015. Improved ancestry estimation for both genotyping and sequencing data using projection Procrustes analysis and genotype imputation. Am J Hum Genet. 96:926–937. 10.1016/j.ajhg.2015.04.018. 26027497 PMC4457959

[iyaf273-B189] Wang C, Zöllner S, Rosenberg NA. 2012. A quantitative comparison of the similarity between genes and geography in worldwide human populations. PLoS Genet. 8:e1002886. 10.1371/journal.pgen.1002886. 22927824 PMC3426559

[iyaf273-B190] Weiss ML . 1991. Saving aboriginal DNA. Science. 253:1467. 10.1126/science.1896851. 1896851

[iyaf273-B191] Weiss GH, Kimura M. 1965. A mathematical analysis of the stepping stone model of genetic correlation. J Appl Probab. 2:129–149. 10.2307/3211879.

[iyaf273-B192] Wendt FR et al 2023. Modeling the longitudinal changes of ancestry diversity in the Million Veteran Program. Hum Genomics. 17:46. 10.1186/s40246-023-00487-3. 37268996 PMC10239111

[iyaf273-B193] Wildschutte JH, Baron A, Diroff NM, Kidd JM. 2015. Discovery and characterization of *Alu* repeat sequences via precise local read assembly. Nucleic Acids Res. 43:10292–10307. 10.1093/nar/gkv1089. 26503250 PMC4666360

[iyaf273-B194] Wohns AW et al 2022. A unified genealogy of modern and ancient genomes. Science. 375:eabi8264. 10.1126/science.abi8264. 35201891 PMC10027547

[iyaf273-B195] Xing J et al 2010. Toward a more uniform sampling of human genetic diversity: a survey of worldwide populations by high-density genotyping. Genomics. 96:199–210. 10.1016/j.ygeno.2010.07.004. 20643205 PMC2945611

[iyaf273-B196] Xu S et al 2009. Genomic dissection of population substructure of Han Chinese and its implication in association studies. Am J Hum Genet. 85:762–774. 10.1016/j.ajhg.2009.10.015. 19944404 PMC2790582

[iyaf273-B197] Xue Y et al 2009. Geographic differences in allele frequencies of susceptibility SNPs for cardiovascular disease. Genetics. 183:1065–1077. 10.1534/genetics.109.107722. 21507254 PMC3103418

[iyaf273-B198] Yang W-Y, Novembre J, Eskin E, Halperin E. 2012. A model-based approach for analysis of spatial structure in genetic data. Nat Genet. 44:725–731. 10.1038/ng.2285. 22610118 PMC3592563

[iyaf273-B199] Yngvadottir B et al 2009. A genome-wide survey of the prevalence and evolutionary forces acting on human nonsense SNPs. Am J Hum Genet. 84:224–234. 10.1016/j.ajhg.2009.01.008. 19200524 PMC2668024

[iyaf273-B200] Zakharia F et al 2009. Characterizing the admixed African ancestry of African Americans. Genome Biol. 10:R141. 10.1186/gb-2009-10-12-r141. 20025784 PMC2812948

[iyaf273-B201] Zhivotovsky LA, Rosenberg NA, Feldman MW. 2003. Features of evolution and expansion of modern humans, inferred from genomewide microsatellite markers. Am J Hum Genet. 72:1171–1186. 10.1086/375120. 12690579 PMC1180270

