# Supplementary material for: Reflections on the Human Genome Diversity Project: a conversation with Marcus W. Feldman, Henry T. Greely, and Mary-Claire King
Source: Genetics. 2026 Feb 12;232(3):iyaf273. doi: 10.1093/genetics/iyaf273 (PMC13017707; doi:10.1093/genetics/iyaf273)
Supplement: iyaf273_Supplementary_Data [file iyaf273_supplementary_data.pdf]

Reflections on the Human Genome Diversity Project: a conversation with Marcus W. Feldman, Henry T. Greely, and Mary-Claire King

Sohini Ramachandran
Noah A. Rosenberg

Online supplementary material

Notes

This section contains additional documentation for the main text. Articles in scientific journals, including news stories in scientific journals, are annotated with author-year citations in this notes section and included in full in the supplementary bibliography below. For other sources, the note contains the full reference. If an item is referenced from the text and from one of the tables, then the full reference is placed in the note associated with the table.

The beginning of the HGDP

"Luca and I were meeting 3 to 4 nights a week for 35 years" — The close collaboration is described by Feldman (2020).

"*Victor McKusick invited us to write a commentary for* Genomics" — McKusick had been one of the founding editors of the journal *Genomics* in 1987 (McKusick & Ruddle 1987).

"*5,000 languages*" — by the time of the 1992 edition, the Ethnologue documented more than 6,000 languages (Grimes 1992).

The planning workshops

"It was a really interesting and intense day" — The agenda for the third HGDP workshop, held at NIH, appears on pages 83-86 of the published Senate hearing (see notes on Table 1 for the full reference).

"$25 million" — An estimate of $25-$30 million for the project cost can be found on p. 3 of an address entitled "The Human Genome Diversity Project" delivered by Luca to a special meeting of UNESCO, September 12, 1994 (https://www.osti.gov/biblio/505328, accessed August 30, 2025).

A Senate hearing

"Collins in his oral testimony" — Collins's oral and written testimony appear on pages 6-11 of the published Senate hearing (see notes on Table 1 for the full reference).

"it would be difficult for the Genome Project to expand its umbrella" — This quotation is from page 22 of the published Senate hearing (see notes on Table 1 for the full reference).

“you’d been considered by NIH Director Bernardine Healy to direct the National Center for Human Genome Research and the Human Genome Project” — see Natalie Angier, “Scientist at work: Mary-Claire King; quest for genes and lost children,” *New York Times*, April 27, 1993. (https://www.nytimes.com/1993/04/27/science/scientist-at-work-mary-claire-king-quest-for-genes-and-lost-children.html, accessed August 30, 2025). This article appeared the day after the HGDP hearing at the United States Senate.

Funding challenges

“project with the Abuelas” —The grant was R01 HG000263, “Sequencing mtDNA for human identification,” and it ran from January 1, 1991 to December 31, 1993 (https://reporter.nih.gov/search/98Lp5r9850qgyZnLfC_88w/project-details/3333330, accessed August 30, 2025). See also King (1991). According to Dolan et al. (2022), 25 ELSI grants had been funded by NCHGR by September 1991 (including this one).

“tells the Chicago Tribune *in 1997*” — The quote from John Moore appears on p. 11 of the *Chicago Tribune*, Sunday, April 27, 1997. Two articles in a two-part series by Paul Salopek “Basically we are all the same” (April 27, 1997) and “Genes offer sampling of hope and fear” (April 28, 1997) appeared as front-page stories. The pages of these articles are available at
https://chicagotribune.newspapers.com/image/168216814/,
https://chicagotribune.newspapers.com/image/168217819/,
https://chicagotribune.newspapers.com/image/168217954/,
https://chicagotribune.newspapers.com/image/168217984/,
https://chicagotribune.newspapers.com/image/168304884/,
https://chicagotribune.newspapers.com/image/168305504/,
https://chicagotribune.newspapers.com/image/168305602/, all accessed June 6, 2025).
The series received the 1998 Pulitzer Prize for Explanatory Reporting.

“does not mention HGDP” — The publication date of the article of Collins, Guyer, & Chakravarti (1997) is November 28, 1997. Author Collins had spoken publicly about HGDP, notably at the Senate hearing on April 26, 1993 (see Table 1 for the full reference). Author Chakravarti is listed on the roster of the National Research Council Committee on Human Genome Diversity, which released its report evaluating HGDP just prior to the Collins et al. (1997) article, on October 21, 1997 (Pennisi 1997).

Interactions of HGDP with Indigenous populations and activists

“slow-motion helicopter followed [O. J. Simpson]” — This event took place on June 17, 1994.

“*isolates of historical interest*” — This phrase appears in the report of the second HGDP workshop (see notes on Table 1 for the full reference).

“*RAFI started a form of criticism*” — A “RAFI communique” entitled “Patents, indigenous peoples, and human genetic diversity” is dated May 1993 (https://www.etcgroup.org/sites/www.etcgroup.org/files/publication/pdf_file/raficom31patents.pdf, accessed August 30, 2025). A RAFI communique entitled “The patenting of human genetic material” dated January/February 1994 refers to the WCIP meeting in Guatemala (https://www.etcgroup.org/sites/www.etcgroup.org/files/publication/492/02/raficom36humangenetic.pdf, accessed August 30, 2025).

“*the HUGO [Human Genome Organisation] ethics committee”* — A “statement on the principled conduct of genetics research” written by the HUGO ELSI committee and referencing the HGDP was reported in Berg et al. (1996).

“the UNESCO [United Nations Educational, Scientific and Cultural Organization] ethics committee” — A report on “bioethics and human population genetics research” written by the UNESCO International Bioethics Committee was reported in 1995 (https://unesdoc.unesco.org/ark:/48223/pf0000132344, accessed August 30, 2025). A news story appeared in *Nature* dated October 5, 1995 (Butler 1995).

“John Moore’s spleen was the first thing that came up” — For more information about the case of John Moore’s spleen, see Annas (1990), Dorney (1990), and Chapter 25 or Skloot (2010). John Moore recounted his version of the events in his testimony before Congress in 1985 (The use of human biological materials in the development of biomedical products: hearing before the Subcommittee on Investigations and Oversight of the Committee on Science and Technology, House of Representatives, Ninety-Ninth Congress, First Session, October 29, 1985. Washington, DC: U. S. Government Printing Office, 1986).

“His dad was a physician in the San Gabriel Valley” — This information about John Moore’s father and how John Moore arrived at UCLA for treatment is from an interview that Hank conducted with John Moore in 1998.

“What kind of cell becomes malignant in hairy cell leukemia” — See Golde (1982).

“The canonical story” — For example, in reference to Moore, Skloot (2010) says “Golde removed his spleen” (p. 199). Annas (1990) says “surgeons removed the spleen”; Dorney (1990) says “Golde recommended that Mr. Moore’s spleen be removed.”

“The best evidence is that the spleen weighed about 6 kg” — Different numbers are cited for the weight of the spleen. John Moore himself used the number 22 pounds in his Congressional testimony in 1985 (p. 241), and Skloot (2010) quotes this number (p. 199). Other numbers can be found; e.g. 14 pounds (Annas 1988) and 16 pounds (Barinaga 1990). In the court decision for Moore’s 1988 appeal, the number 6 kg (13.2 pounds) is cited in a “Case history” paragraph that likely summarizes Moore’s medical records (Moore v. Regents of University of California, Court of Appeal, Second District, Division 4, California, B021195, decided July 21, 1988; https://caselaw.findlaw.com/court/ca-court-of-appeal/1759449.html, accessed August 31, 2025). Hank conducted an interview with Dr. Golde in 1998; his notes from that interview record that Golde told him that the pathology report said “over 6,000 grams,” and that Golde said that he thought it weighed 18 pounds.

“He discovers the world's second known human retrovirus in this cell line, HTLV-2” — See Blakeslee (1984).

“the poor American anthropologist” — This reference is to anthropologist Carol Jenkins; see Taubes (1995) and Lehrman (1996). Taubes notes the role of the 1990s expansion of internet use in the distortion of Jenkins’s work: “By distributing the release via the Internet — a medium prized by scientists for its ability to disseminate information, but one proving equally adept at spreading misinformation — RAFI ensured a wide and rapid airing.”

"put out a press release" — In the RAFI communique entitled "New questions about management and exchange of human tissues at NIH; indigenous person's cells patented" dated March/April 1996, it is mentioned that the press release of October 4, 1995 linked the HGDP to the Hagahai patent incident (https://www.etcgroup.org/sites/www.etcgroup.org/files/publication/468/01/raficom48nih.pdf, accessed August 30, 2025). Text from the October 4, 1995 press release is quoted by Cunningham (1998).

Model ethical protocol

"I had a law student write a nice paper" — the reference is: Amy Knight Burns, "The paradox of autonomy: navigating the control of genetic information" (October 8, 2011). Available at SSRN: https://doi.org/10.2139/ssrn.1941049.

Hosting the cell lines at CEPH

"In order to enable matching by HLA type for organ transplants" — See Dausset (1981) and Mandel et al. (2009).

"Howard Cann had been professor of pediatrics and genetics at Stanford" — See CEPH's page about Howard Cann (https://cephb.fr/en/presentation_howard_cann.php, accessed August 30, 2025).

Achievements of the HGDP in relation to its funding

"just one 10th of one percent of the Human Genome Project" — The cost of the Human Genome Project is commonly quoted at $2.7 billion, as in the September 29, 2022 *Wired* story by Emily Mullin "The era of fast, cheap genome sequencing is here" (https://www.wired.com/story/the-era-of-fast-cheap-genome-sequencing-is-here/, accessed August 30, 2025). The National Human Genome Research Institute Human Genome Project fact sheet reports an estimate of $3 billion (https://www.genome.gov/about-genomics/educational-resources/fact-sheets/human-genome-project, accessed August 30, 2025.)

Coda

"HGDP was a huge contribution" — The *Stanford Report* obituary referenced is by Amy Adams and Hanae Armitage, "Luigi Luca Cavalli-Sforza, a giant in population genetics and professor emeritus, dies at 96," September 10, 2018 (https://med.stanford.edu/news/all-news/2018/09/luigi-luca-cavalli-sforza-a-giant-in-population-genetics-dies-at-96.html, accessed August 30, 2025).

"the cell lines… are still there" — See https://cephb.fr/en/hgdp_panel.php; for inquiries, contact HGDP_Manager@fjd-ceph.org.

Appendix

"HGDP-CEPH Melanesian and Papuan data" — The document describing the Nobel Prize in Physiology or Medicine to Svante Pääbo, referencing the HGDP-CEPH Melanesians and Papuans and data of Li et al. (2008), is located at https://www.nobelprize.org/uploads/2022/10/advanced-medicineprize2022.pdf (accessed September 9, 2025).

Table 1

"NSF grant of $178,178" — See the notes on Table 3.

"Workshop 1" — The report is available at https://www.osti.gov/biblio/505330 (accessed August 30, 2025).

"Workshop 2" — The report is available at https://www.osti.gov/biblio/505329 (accessed August 30, 2025).

"Workshop 3" — The report is available at https://www.osti.gov/biblio/505328 (accessed August 30, 2025)

"HGDP hearing" — Human Genome Diversity Project: Hearing before the Committee on Governmental Affairs, United States Senate, One Hundred Third Congress, First Session. Washington, DC: U. S. Government Printing Office. ISBN 0-16-043334-7.

"Workshop 4" — The report is available at https://www.osti.gov/biblio/505331 (accessed August 30, 2025).

"WCIP meeting" — This meeting is described by Kahn (1994).

"MacArthur Foundation grant" — See the notes on Table 3.

Table 2

"Luca Cavalli-Sforza enrolls at the University of Turin, later transferring to the University of Pavia" — See Stone & Lurquin (2005, p. 26) and Feldman (2020).

"Cavalli-Sforza goes to Cambridge to study with R. A. Fisher" — See Stone & Lurquin (2005, p. 38), Feldman (2020), and Edwards (2021).

"Curt Stern retires from the University of California, Berkeley" — See Neel (1983).

"Cavalli-Sforza joins the faculty of the Stanford Department of Genetics, having been recruited by Joshua Lederberg" — See Feldman (2020).

"Marc Feldman joins the faculty of the Stanford Department of Biological Sciences" — See Feldman (2020).

"Ruth Kirschstein becomes Director of the National Institute for General Medical Sciences" — See the 2012 NIGMS Feedback Loop Blog post by Judith Greenberg https://nigms.nih.gov/loop/2012/02/remarkable-ruth-kirschstein-new-biography-of-an-nigms-director, accessed August 30, 2025).

"John Moore's spleen is removed" — See Annas (1990), Dorney (1990), and Skloot (2010, p. 199).

"Mary-Claire King begins working with the Abuelas de Plaza de Mayo" — See King (1991).

"Judith Greenberg becomes Director of the Division of Genetics and Developmental Biology" — See the 2015 NIGMS Feedback Loop Blog post by Jon Lorsch https://nigms.nih.gov/loop/2015/01/judith-greenberg-named-deputy-director-of-nigms, accessed August 30, 2025).

"The California Supreme Court rules that John Moore does not have rights to profits" — See Annas (1990), Dorney (1990), and Skloot (2010, p. 205).

"Stanford hosts a Centennial Symposium" — Greely (2021) describes this symposium on p. 296. The event date, associated with the centennial of the university's opening in 1891, is noted by the Stanford University archives (https://archives.stanford.edu/catalog/sc1031_aspace_ref223_1sw, accessed August 30, 2025).

"Bernardine Healy becomes Director of the National Institutes of Health" — See the NIH Almanac post (https://www.nih.gov/about-nih/nih-almanac/bernadine-healy-md, accessed August 30, 2025).

"Allan Wilson (1934-1991) dies of leukemia (July 21, 1991)" — See Cann (1993).

"Francis Collins becomes Director of the National Center for Human Genome Research" — For the start date, see the NHGRI Human Genome Project timeline (https://www.genome.gov/human-genome-project/timeline, accessed August 30, 2025); fo the end date, see an NHGRI press release (https://www.genome.gov/27026551/2008-release-francis-s-collins-to-step-down-as-director-of-national-human-genome-research-institute, accessed August 30, 2025).

"Myriad Genetics applies for patent protection" — See Sherkow et al. (2024).

"A patent is awarded" — The patent number was 5,397,696, dated March 14, 1995 (https://patentimages.storage.googleapis.com/6a/96/65/94b964ed809b40/US5397696.pdf, accessed August 30, 2025). See Taubes (1995) and Lehrman (1996) for further information about the Hagahai patent incident.

"The legislation known as the Clinton health care plan" — The date of this event is September 26, 1994.

"Craig Venter announces a plan" — See Putman (1998).

"The draft sequence of the human genome is reported" — This event took place on June 26, 2000; see the NHGRI Human Genome Project timeline (https://www.genome.gov/human-genome-project/timeline, accessed August 30, 2025). A video of the event appears at https://www.youtube.com/watch?v=slRyGLmt3qc (accessed August 30, 2025).

"The International Haplotype Map Project is launched" — See Dove (2002).

"Luca Cavalli-Sforza (1922-2018) dies" — See the *Stanford Report* obituary https://med.stanford.edu/news/all-news/2018/09/luigi-luca-cavalli-sforza-a-giant-in-population-genetics-dies-at-96.html (accessed August 30, 2025). See also Feldman (2020) and Edwards (2021).

Table 3

The information on the grants in the table is available as follows (all websites accessed August 30, 2025).
Feldman: https://www.nsf.gov/awardsearch/showAward?AWD_ID=9209745;
Greely: https://www.nsf.gov/awardsearch/showAward?AWD_ID=9610371;
Carter: https://www.nsf.gov/awardsearch/showAward?AWD_ID=9610336;
Weiss: https://www.nsf.gov/awardsearch/showAward?AWD_ID=9610098;

Kwok: https://www.nsf.gov/awardsearch/showAward?AWD_ID=9610342;
Thomson: https://www.nsf.gov/awardsearch/showAward?AWD_ID=9610505;
Shriver: https://www.nsf.gov/awardsearch/showAward?AWD_ID=9610332,
https://www.nsf.gov/awardsearch/showAward?AWD_ID=9796180, and
https://www.nsf.gov/awardsearch/showAward?AWD_ID=9996298;
Batzer: https://www.nsf.gov/awardsearch/showAward?AWD_ID=9610147;
Jorde: https://www.nsf.gov/awardsearch/showAward?AWD_ID=9700729.

The transfer of $38,000 to NSF from NCHGR is documented on p. 147 of the “Briefing book for the heading on the Human Genome Diversity Project, Committee on Governmental Affairs, United States Senate” (https://archive.genome.gov/repositories/2/digital_objects/19, accessed August 30, 2025); p. 9 of this document indicates contributions of NIGMS and DOE.

The MacArthur Foundation grant is described at https://www.macfound.org/grantee/stanford-university-morrison-institute-for-population-and-resource-studies-5457/ (accessed August 30, 2025).

Table 4

The first HGDP workshop report from the meeting held at Stanford in July 1992 is available at https://www.osti.gov/biblio/505330 (accessed August 30, 2025).

Supplementary references

Annas GJ (1988). Whose waste is it anyway? The case of John Moore. *Hastings Center Report* 18: 37-39. https://doi.org/10.2307/3562222.

Annas GJ (1990). Outrageous fortune: selling other people’s cells. *Hastings Center Report* 20: 36-39. https://doi.org/10.2307/3563426.

Barinaga M (1990). A muted victory for the biotech industry. *Science* 249: 239. https://doi.org/10.1126/science.2374922.

Berg K, Capron A, Chadwick RF, Kirby K, Knoppers BM, Macer DRJ, McKusick VA, Muller-Hill B, Murray TH, Niermeijerm MF, Pompidou A, Rodota S, Takebe H, Wexler N (1996). Statement on the principled conduct of genetics research (HUGO code of ethics). *Eubios Journal of Asian and International Bioethics* 6: 59-60. https://www.eubios.info/HUGO.htm.

Blakeslee S (1984). Patient sues for title to own cells. *Nature* 311: 198. https://doi.org/10.1038/311198a0.

Butler D (1995). Genetic diversity proposal fails to impress international ethics panel. *Nature* 377: 373. https://doi.org/10.1038/377373a0.

Cann RL (1993). Obituary: Allan C. Wilson, 1935-1991. *Human Biology* 65: 343-358. http://www.jstor.org/stable/41464858.

Cunningham H (1998). Colonial encounters in postcolonial contexts: patenting indigenous DNA and the Human Genome Diversity Project. *Critique of Anthropology* 18: 205-233. https://doi.org/10.1177/0308275X9801800205.

Dolan DD, Lee SSJ, Cho MK (2022). Three decades of ethical, legal, and social implications research: looking back to chart a path forward. *Cell Genomics* 2: 100-150. https://doi.org/10.1016/j.xgen.2022.100150.

Dorney MS (1990). Comment: Moore v. The Regents of the University of California: balancing the need for biotechnology innovation against the right of informed consent. *High Technology Law Journal* 5: 333-369. https://www.jstor.org/stable/24122359.

Dove A (2022). Mapping project moves forward despite controversy. *Nature Medicine* 8: 1337. https://doi.org/10.1038/nm1202-1337a.
Dausset J (1981). The major histocompatibility complex in man. *Science* 213: 1469-1474. https://doi.org/10.1126/science.6792704.
Feldman MW (2020). L. Luca Cavalli-Sforza: a Renaissance scientist. *Theoretical Population Biology* 133: 75-79. https://doi.org/10.1016/j.tpb.2019.11.009.
Edwards AWF (2021). Luigi Luca Cavalli-Sforza, 25 January 1922 – 31 August 2018. *Biographical Memoirs of Fellows of the Royal Society* 70: 79-105. https://doi.org/10.1098/rsbm.2020.0015.
Golde DW (1982). Therapy of hairy-cell leukemia. *New England Journal of Medicine* 307: 495-496. https://doi.org/10.1056/NEJM198208193070809.
Greely HT (2021). *CRISPR People*. Cambridge, MA: MIT Press.
Grimes BF (ed.) (1992). *Ethnologue: Languages of the World*, volume 12. Dallas: Summer Institute of Linguistics.
Kahn P (1994). Genetic diversity project tries again. *Science* 266: 720-722. https://doi.org/10.1126/science.7973621.
King M-C (1991). An application of DNA sequencing to a human rights problem. *Molecular Genetic Medicine* 1: 117-131. https://doi.org/10.1016/B978-0-12-462001-8.50009-0.
Lehrman S (1996). US drops patent claim to Hagahai cell line. *Nature* 384: 500. https://doi.org/10.1038/384500a0.
Mandel J-L, Lathrop M, Cann HM (2009). Jean B Dausset, 19 October 1916 - 6 June 2009. *European Journal of Human Genetics* 17: 1365-1366. https://doi.org/10.1038/ejhg.2009.150.
McKusick VA, Ruddle FH (1987). A new discipline, a new name, a new journal. *Genomics* 1: 1–2. https://doi.org/10.1016/0888-7543(87)90098-X.
Neel JV (1983). Curt Stern, 1902-1981. *Annual Review of Genetics* 17: 1-10. https://doi.org/10.1146/annurev.ge.17.120183.000245.
Pennisi E (1997). NRC OKs long-delayed survey of human genome diversity. *Science* 278: 568. https://doi.org/10.1126/science.278.5338.568.
Putman L (1998). US firm claims it can “sequence human genome in 3 years.” *Lancet* 351: 1566. https://10.1016/S0140-6736(05)61137-3.
Sherkow JS, Cook-Deegan R, Greely HT (2024). The Myriad decision at 10. *Annual Review of Genomics and Human Genetics* 25: 397-419. https://10.1146/annurev-genom-010323-011239.
Skloot R (2010). *The Immortal Life of Henrietta Lacks*. New York: Crown.
Taubes G (1995). Scientists attacked for ‘patenting' Pacific tribe. *Science* 270: 1112. https://doi.org/10.1126/science.270.5239.1112.
